# SPHERIOUSLY? The challenges of estimating sphere radius non-invasively in the human brain from diffusion MRI

**DOI:** 10.1016/j.neuroimage.2021.118183

**Published:** 2021-08-15

**Authors:** Maryam Afzali, Markus Nilsson, Marco Palombo, Derek K Jones

**Affiliations:** a Cardiff University Brain Research Imaging Centre (CUBRIC), School of Psychology, Cardiff University, Cardiff, United Kingdom; b Clinical Sciences Lund, Radiology, Lund University, Lund, Sweden; c Centre for Medical Image Computing, Department of Computer Science, University College London, London, United Kingdom

**Keywords:** Diffusion-weighted imaging, Direction-averaged diffusion signal, b-tensor encoding, Three-compartment model, Spherical compartment

## Abstract

The Soma and Neurite Density Imaging (SANDI) three-compartment model was recently proposed to disentangle cylindrical and spherical geometries, attributed to neurite and soma compartments, respectively, in brain tissue. There are some recent advances in diffusion-weighted MRI signal encoding and analysis (including the use of multiple so-called ’b-tensor’ encodings and analysing the signal in the frequency-domain) that have not yet been applied in the context of SANDI. In this work, using: (i) ultra-strong gradients; (ii) a combination of linear, planar, and spherical b-tensor encodings; and (iii) analysing the signal in the frequency domain, three main challenges to robust estimation of sphere size were identified: First, the Rician noise floor in magnitude-reconstructed data biases estimates of sphere properties in a non-uniform fashion. It may cause overestimation or underestimation of the spherical compartment size and density. This can be partly ameliorated by accounting for the noise floor in the estimation routine. Second, even when using the strongest diffusion-encoding gradient strengths available for human MRI, there is an *empirical* lower bound on the spherical signal fraction and radius that can be detected and estimated robustly. For the experimental setup used here, the lower bound on the sphere signal fraction was approximately 10%. We employed two different ways of establishing the lower bound for spherical radius estimates in white matter. The first, examining power-law relationships between the DW-signal and diffusion weighting in empirical data, yielded a lower bound of 7μm, while the second, pure Monte Carlo simulations, yielded a lower limit of 3μm and in this low radii domain, there is little differentiation in signal attenuation. Third, if there is sensitivity to the transverse intra-cellular diffusivity in cylindrical structures, e.g., axons and cellular projections, then trying to disentangle two diffusion-time-dependencies using one experimental parameter (i.e., change in frequency-content of the encoding waveform) makes spherical radii estimates particularly challenging. We conclude that due to the aforementioned challenges spherical radii estimates may be biased when the corresponding sphere signal fraction is low, which must be considered.

## 1 Introduction

Diffusion magnetic resonance imaging (dMRI) is a non-invasive technique widely used to study brain microstructure *in vivo*. Most dMRI methods are based on the conventional Stejskal-Tanner experiment (Stejskal and Tanner, 1965) that applies a pair of pulsed field gradients along a single axis for each signal preparation, which we refer to here as ’linear’ encoding. Using linear encoding, disentangling different microstructural properties such as their size, shape, and orientation is far from trivial (Lampinen, Szczepankiewicz, Mårtensson, van Westen, Sundgren, Nilsson, 2017, Novikov, Fieremans, Jespersen, Kiselev, 2019). Such features may be entangled in the encoding process resulting in low specificity in their estimation. This is particularly problematic in dMRI where the image voxel is on the scale of a millimeter, and can therefore contain multiple microenvironments.

Biophysical modeling is often used to tackle the inverse problem of inferring relevant tissue features (such as cell size, shape, and orientation) from the measured dMRI signal (Assaf, Blumenfeld-Katzir, Yovel, Basser, 2008, Mitra, Sen, Schwartz, Le Doussal, 1992, Pasternak, Sochen, Gur, Intrator, Assaf, 2009, Stanisz, Wright, Henkelman, Szafer, 1997, Wiegell, Larsson, Wedeen, 2000, Zhang, Schneider, Wheeler-Kingshott, Alexander, 2012). Most contemporary dMRI models for neural tissue share some common assumptions and features. First, they separate the tissue into intra- and extra- neurite compartments. Second, the exchange between the compartments is considered to be negligible, such that each compartment has a fixed and time-invariant signal fraction $f_{i}$, where $\sum_{i}f_{i}=1$. Third, most models treat the intra-neurite compartment as a ’stick’ - that is a compartment in which the diffusivity perpendicular to the long axis of the compartment is assumed to be effectively zero. This assumption is based on the lack of sensitivity to the neurite diameter (Nilsson et al., 2017). Different models have been used for the orientation dispersion of the compartments, represented by an orientation distribution function (ODF). Some models consider only purely parallel orientations (i.e., a delta function on the sphere ODF) (Assaf, Blumenfeld-Katzir, Yovel, Basser, 2008, Stanisz, Wright, Henkelman, Szafer, 1997) while others use spherical harmonics (Jespersen et al., 2007) or a function such as the Watson distribution (Zhang et al., 2012) to characterise orientation dispersion. A Gaussian anisotropic representation is most often used for the extra-neurite compartment. Its orientation is determined by the mean of the fiber ODF and is characterized by axial and radial diffusivities. The extra-axonal component in both white and gray matter is believed to model all non-axonal water including that in the soma. Recent studies have shown that the two-compartment model is not a good representation of the signal in gray matter (Afzali, Aja-Fernández, Jones, 2020, Afzali, Pieciak, Newman, Garifallidis, Özarslan, Cheng, Jones, 2020, Henriques, Jespersen, Shemesh, 2019, Jespersen, Olesen, Ianuş, Shemesh, 2019, McKinnon, Jensen, Glenn, Helpern, 2017, Palombo, Shemesh, Ianus, Alexander, Zhang, 2018, Veraart, Fieremans, Novikov, 2019). This can be due to non-negligible water exchange processes occurring between intra- and extra-cellular compartments and between different intracellular compartments (Jelescu, Novikov, 2020, Veraart, Fieremans, Rudrapatna, Jones, Novikov, 2018), or the assumption that the water inside the soma behaves the same as water in extracellular space (Palombo, Shemesh, Ianus, Alexander, Zhang, 2018, Palombo, Shemesh, Ianus, Alexander, Zhang, 2018). Addressing this model insufficiency, Palombo et al. (Palombo et al., 2020) first demonstrated with non-trivial numerical simulations that, under specific experimental conditions, the contribution of soma to the total intracellular dMRI signal can be disentangled from that of neurite, and then introduced a three-compartment model called Soma And Neurite Density Imaging (SANDI), which decomposes the measured dMRI signal into three main sources: extra-cellular space, neurite and soma. If there is any sensitivity to the size of a compartment, the diffusion MRI signal in that compartment will have a time-dependence.

The inverse problem that arise when using complex multi-compartment models to infer microstructural information from the diffusion-weighted signal can be highly ill-posed and give rise to degeneracies in the model-parameter estimation, i.e., in the forward sense, completely different sets of model parameters predict the same dMRI signals (Jelescu, Veraart, Fieremans, Novikov, 2016, Jones, Knösche, Turner, 2013, Lampinen, Szczepankiewicz, Mårtensson, van Westen, Hansson, Westin, Nilsson, 2020, Lampinen, Szczepankiewicz, Mårtensson, van Westen, Sundgren, Nilsson, 2017, Lampinen, Szczepankiewicz, Novén, van Westen, Hansson, Englund, Mårtensson, Westin, Nilsson, 2019, Novikov, Kiselev, Jespersen, 2018). SANDI, like other multi-compartment models (Jelescu, Veraart, Fieremans, Novikov, 2016, Lampinen, Szczepankiewicz, Novén, van Westen, Hansson, Englund, Mårtensson, Westin, Nilsson, 2019), may suffer from the same degeneracy problems.

The degeneracy problem is coupled with the strategy used for diffusion encoding. In single diffusion encoding (SDE), the MR signal is sensitized to diffusion using a pair of gradient pulses that encode the position of the spins along the axis defined by the diffusion gradients. Double diffusion encoding (DDE) contains two pairs of pulsed-field gradients that are separated from each other with a mixing time τ (Callaghan, 2011, Cory, Garroway, Miller, 1990, Shemesh, Cohen, 2011, Shemesh, Jespersen, Alexander, Cohen, Drobnjak, Dyrby, Finsterbusch, Koch, Kuder, Laun, et al., 2016). This approach has been utilized by several groups for extracting microstructure information (Benjamini, Komlosh, Basser, Nevo, 2014, Coelho, Pozo, Jespersen, Jones, Frangi, 2019, Ianuş, Drobnjak, Alexander, 2016, Jespersen, Lundell, Sønderby, Dyrby, 2013, Özarslan, Shemesh, Basser, 2009, Yang, Tian, Leuze, Wintermark, McNab, 2018). Correlation Tensor MRI (CTI) is able to disentangle the isotropic and anisotropic kurtosis components arising from restricted diffusion. However, it does not model the microstructural features directly (Henriques et al., 2020). A framework called q-space trajectory imaging (QTI) was recently introduced by (Westin et al., 2016) to probe tissue using different gradient waveforms. The traditional, pulsed field gradient sequences attempt to probe a point in q-space but in q-space trajectory encoding, time-varying gradients are used to probe a trajectory in q-space. The effect of the encoding waveform can be analyzed using the b-tensor (Topgaard, 2017, Westin, Szczepankiewicz, Pasternak, Özarslan, Topgaard, Knutsson, Nilsson, 2014, Westin, Knutsson, Pasternak, Szczepankiewicz, Özarslan, van Westen, Mattisson, Bogren, O’donnell, Kubicki, et al., 2016). In this framework, SDE is a special realization of linear tensor encoding (LTE) where the b-tensor has only one non-zero eigenvalue as all gradients are applied along the same axis. DDE is a special case of planar tensor encoding (PTE) as all gradients lie on a plane and the b-tensor has two non-zero eigenvalues. In spherical tensor encoding (STE) the gradients may point in all directions giving rise to a rank-3 b-matrix. Recently, b-tensor encoding has been used to resolve the degeneracy problem (Coelho, Pozo, Jespersen, Jones, Frangi, 2019, Fieremans, Veraart, Benjamin, Filip, Nilsson, Novikov, 2018, Gyori, Clark, Dragonu, Alexander, Kaden, 2019, Lampinen, Szczepankiewicz, Mårtensson, van Westen, Hansson, Westin, Nilsson, 2020, Reisert, Kiselev, Dhital, 2019). While these studies have shown that considering the b-tensor provides an improvement in the accuracy of parameter estimates, the time-dependence of the diffusion-weighted signal can be used as another feature to add information. In particular, Gyori et al. (Gyori et al., 2019) recently proposed a method based on a three-compartment model to estimate neurite and soma features (e.g. signal fractions and intra-compartment apparent diffusivities) from combined LTE and STE data. Notably, Gyori et al. treated the signal coming from the spherical compartment as a simple mono-exponential with a fixed, time-invariant small diffusivity. This implicitly assumes that the spherical compartment would show the same signal behavior for linear and spherical tensor encoding for a given b-value. However, Lundell et al. (Lundell et al., 2019) demonstrated that this is only true in restricted geometries when the LTE and STE waveforms have the same frequency power spectra. The importance of time dependence for encoding going beyond SDE has also been considered in the context of STE (Jespersen et al., 2019) and DDE (Henriques et al., 2020).

In this study, we applied b-tensor encoding with variable power spectra (including LTE and STE waveforms that were not spectrally matched to each other) to investigate whether and how it improves fitting of the SANDI model. We exploited all three forms of b-tensor, i.e., LTE, STE, and PTE. As a signal model, we adopted van Gelderen’s model of the spherical compartment (Vangelderen et al., 1994), which explicitly includes both diffusion gradient pulse width and separation (Δ and δ). The challenge in using the free gradient waveforms, however, is that Δ and δ are poorly defined, and so the time-dependency of the obtained signal is not well-defined in the time domain. Therefore, to find a closed-form for the diffusion-weighted signal decay in the spherical compartment, we adopted this model to the frequency domain (Lundell, Nilsson, Dyrby, Parker, Cristinacce, Zhou, Topgaard, Lasič, 2019, Nilsson, Lasič, Drobnjak, Topgaard, Westin, 2017, Stepišnik, 1993). The main findings of this paper are as follows:

• **Noise Sensitivity:** Even when complementing LTE with STE- and PTE-data, fitting the spherical radii properties remains challenging (when the sphere signal fraction is small, i.e. ≤10%), with simulations showing biases in parameter estimates. Here we demonstrate that it is predominantly the Rician distribution of the noise (Gudbjartsson, Patz, 1995, Koay, Özarslan, Basser, 2009) (and associated noise floor) that impacts the estimation of spherical compartment properties. Such biases disappear when simulating purely Gaussian noise). However, if the Rician noise floor is accounted for in the model-fitting (albeit naively) much of the noise-floor induced bias is ameliorated.
• **Lower Bound on Sphere Signal Fraction:** By using the F-statistic to compare nested models (i.e., those that do or do not include a sphere fraction) in simulated data where the spherical radii properties are varied systematically, it was possible to identify a lower bound on the detectable MRI sphere signal fraction limit. This was around 10% for data with SNR = 50.
• **Lower Bound on Sphere Radius:** The empirical lower limit on sphere radius in brain tissue was estimated by comparing exponents in power-law relationships between the dMRI signal and b-value fitted to simulated data, with exponents observed empirically *in vivo*. We employed two different ways of establishing the lower bound for spherical radius estimates in white matter. The first, examining power-law relationships between the DW-signal and diffusion weighting in empirical data, yielded a lower bound of 7μm, while the second, pure Monte Carlo simulations, yielded a lower limit of 3μm. In addition, there is little differentiation in signal attenuation for low radii spheres (e.g. $R_{\text{sphere}}<4\mu m$, Fig 3).
• **One or two time-dependent components:** In addition to the challenge of estimating sphere size, the fitting becomes even more challenging if we have cylinders instead of sticks. As shown by Veraart et al. (Veraart et al., 2020), at 300 mT/m, and with appropriate diffusion times, we have sensitivity to the internal perpendicular diffusivity in cylindrical pores (demonstrated by a break from a power-law relationship between signal intensity and b-value). A challenge then arises when trying to disentangle two time-dependencies by varying the same experimental parameter (i.e, changing the frequency content of the gradient-encoding waveform). With currently-available pipelines, this prevents reliable estimates of sphere radii in white matter when there is sensitivity to intra-axonal radial diffusivity, and indeed may plague grey matter modelling if there is sensitivity to water in the astrocytic processes. We should note that most of the cellular projections are smaller than 3 microns in radius, while the majority of soma are above 3 microns (Di Benedetto, Malik, Begum, Jablonowski, Gómez-González, Neumann, Rupprecht, 2016, Fannon, Tarmier, Fulton, 2015, Mohamed, Paisley, Meyer, Bigbee, Sato-Bigbee, 2020, Papageorgiou, Gabriel, Fetani, Kann, Heinemann, 2011, Savtchenko, Bard, Jensen, Reynolds, Kraev, Medvedev, Stewart, Henneberger, Rusakov, 2018, Zhang, Bassam, Thomas, Williams, Liu, Nance, Rojas, Slusher, Kannan, 2016). Therefore, the ambiguity here is more relevant to WM voxels given a low soma density there.

## 2 Theory

Multi-compartment models express the diffusion-weighted signal as the sum of several compartments.

(1) $$S=\sum_{k}f_{k}S_{k}$$

where $f_{k}$ is the signal fraction ($\sum_{k}f_{k}=1$) and $S_{k}$ is the signal from the kth compartment.

For a general **B**-tensor, the diffusion-weighted MR signal is modeled as:

(2) $$\begin{array}{ccc} S\left(B\right)/S_{0} & = & f_{\mathrm{cylinder}}\int_{S^{2}}W\left(\kappa ,n\right)e^{-B:D_{\text{cylinder}}\left(n,t\right)}dn\\ & & +f_{\text{sphere}}S_{\text{sphere}}\left(D_{\text{sphere}}\left(t\right),B\right)+f_{\text{ball}}e^{-bD_{\text{ball}}} \end{array}$$

where $f_{\mathrm{cylinder}}$, $f_{\mathrm{ball}}$, $f_{\mathrm{sphere}}$, $D_{\mathrm{cylinder}}\left(n,t\right)=\left(D_{\mathrm{in}}^{||}-D_{\mathrm{in}}^{⊥}\left(t\right)\right)nn^{T}+D_{\mathrm{in}}^{⊥}\left(t\right)I$, $D_{\mathrm{ball}}$ and $D_{\mathrm{sphere}}$ are the cylinder, ball and sphere signal fractions and diffusivities, respectively (Murday and Cotts, 1968). W(n) is the Watson orientation distribution function (ODF) and κ is the dispersion parameter. The sphere component models the soma, the ball component models the extra cellular compartment, and the cylinder component models the neurites (Palombo et al., 2020). The diffusion weighting tensor B is given by $B=\int_{0}^{TE}q\left(t\right)q^{⊺}\left(t\right)\text{dt}$ where $q\left(t\right)=\gamma \int_{0}^{t}g\left(t^{\prime }\right){\text{dt}}^{\prime }$ (Eriksson, Lasič, Nilsson, Westin, Topgaard, 2015, Westin, Szczepankiewicz, Pasternak, Özarslan, Topgaard, Knutsson, Nilsson, 2014, Westin, Knutsson, Pasternak, Szczepankiewicz, Özarslan, van Westen, Mattisson, Bogren, O’donnell, Kubicki, et al., 2016), and γ is the gyromagnetic ratio. Axial and radial elements in the diagonal axisymmetric b-tensor are $b_{||}$ and $b_{⊥}$ respectively, b-value, b is the trace of B and $b_{\Delta }=\left(b_{||}-b_{⊥}\right)/b$. For linear (LTE), planar (PTE) and spherical (STE) tensor encoding, $b_{\Delta }=1$, −1/2, and 0 respectively (Eriksson et al., 2015).

To remove the effect of fiber orientation dispersion (Jespersen, Lundell, Sønderby, Dyrby, 2013, Lasič, Szczepankiewicz, Eriksson, Nilsson, Topgaard, 2014), the acquired signal is averaged over all diffusion directions for each shell. This so-called ’powder-averaged’ signal (Callaghan, Jolley, Lelievre, 1979, Edén, 2003) has less complexity than the orientation-dependent signal, and yields a signal whose orientationally-invariant aspects of diffusion are preserved but with an orientation distribution that mimics complete dispersion of anisotropic structures. Compartmental diffusion is represented with axisymmetric diffusion tensors which are described by isotropic diffusivity, $D_{I}=1/3D_{||}+2/3D_{⊥}$, and anisotropy, $D_{\Delta }=\left(D_{||}-D_{⊥}\right)/\left(D_{||}+2D_{⊥}\right)$ where $D_{||}$ and $D_{⊥}$ are the axial and radial diffusivities, respectively. $D_{\Delta }$ changes between −1/2 for a planar tensor to 1 for a stick. The signal attenuation from the kth compartment is given by (Eriksson, Lasič, Nilsson, Westin, Topgaard, 2015, Lampinen, Szczepankiewicz, Novén, van Westen, Hansson, Englund, Mårtensson, Westin, Nilsson, 2019):

(3) $$\begin{array}{ccc} A_{k}\left(b,b_{\Delta },D_{I;k},D_{\Delta ;k}\right) & = & \exp(-bD_{I;k}\left[1-b_{\Delta }D_{\Delta ;k}\right])\cdot \\ & & g(3bD_{I;k}b_{\Delta }D_{\Delta ;k}) \end{array}$$

where

(4) $$g\left(\alpha \right)=\int_{0}^{1}\exp\left(-\alpha x^{2}\right)dx=\sqrt{\frac{\pi }{4\alpha }}\text{erf}\left(\sqrt{\alpha }\right)$$

and erf(.) is the error function (Callaghan et al., 1979). Diffusion inside the sphere and ball is isotropic ($D_{\Delta ;\text{sphere}}=0$, $D_{\Delta ;\text{ball}}=0$) while for the cylinder and stick it is anisotropic ($D_{\Delta ;\text{cylinder}}>0$, $D_{\Delta ;\text{stick}}=1$). Therefore, the full signal equation is given by:

(5) $$\begin{array}{ccc} S/S_{0} & = & f_{\text{cylinder}/\text{stick}}A_{\text{cylinder}/\text{stick}}+f_{\text{sphere}}A_{\text{sphere}}\\ & & +f_{\text{ball}}A_{\text{ball}} \end{array}$$

### 2.1 Two and three-compartment models

#### 2.1.1 Cylinder + Ball + Sphere (Extended SANDI model)

In the original SANDI framework, the dMRI signal in brain tissue is assumed to arise from three main non-exchanging compartments: (i) intra-neurite (modeled as diffusion in sticks); (ii) intra-soma (modeled as diffusion constrained to a sphere); and (iii) extra-cellular (modeled as isotropic Gaussian diffusion). Here we extend this model to consider the perpendicular diffusivity in the intra-neurite compartment, $D_{\text{cylinder}}^{⊥}\left(t\right)$, thereby modeling it with cylinders instead of sticks. We additionally explore the feasibility of modeling the intra-axonal perpendicular diffusivity and the additive spherical, $D_{\text{sphere}}\left(t\right)$, sensitivity simultaneously.

(6) $$S/S_{0}=f_{\text{cylinder}}A_{\text{cylinder}}+f_{\text{sphere}}A_{\text{sphere}}+f_{\text{ball}}A_{\text{ball}}$$

For complex gradient waveforms, the diffusion time is ill-defined. We therefore consider the diffusion spectrum $D_{\text{cylinder}}^{⊥}\left(\omega \right)$, $D_{\text{sphere}}\left(\omega \right)$ (Lundell, Nilsson, Dyrby, Parker, Cristinacce, Zhou, Topgaard, Lasič, 2019, Stepišnik, 1993) in our analyses of compartment size.

The restricted DW-signal inside the sphere and cylinder is S=exp(−ρ) where ρ is (Lundell, Nilsson, Dyrby, Parker, Cristinacce, Zhou, Topgaard, Lasič, 2019, Stepišnik, 1993):

(7) $$\rho =\frac{1}{2\pi }\int_{-\infty }^{\infty }f^{T}\left(\omega \right)D\left(\omega \right)f\left(-\omega \right)d\omega$$

where $f\left(\omega \right)=\int_{0}^{\tau }f\left(t\right)e^{-i\omega t}\text{dt}$, $f\left(t\right)=\gamma \int_{0}^{t}g\left(t^{\prime }\right){\text{dt}}^{\prime }$, g(t) is the gradient waveform. D(ω) can be expressed with a rotation matrix R as $D\left(\omega \right)=R\Lambda \left(\omega \right)R^{-1}$ where Λ(ω) is the diagonal matrix containing diffusion spectra $\lambda_{j}\left(\omega \right)$ along the restriction principal axes. The analytical expression for $\lambda_{j}\left(\omega \right)$ in the case of restricted diffusion in planar, cylindrical and spherical geometries, is the weighted sum of negative Lorentzians:

(8) $$\lambda_{j}\left(\omega \right)=\sum_{i}B_{i}\frac{a_{i}D_{0}\omega^{2}}{a_{i}^{2}D_{0}^{2}+\omega^{2}}$$

where for a cylinder, $\lambda_{1}\left(\omega \right)=\lambda_{2}\left(\omega \right)=\lambda \left(\omega \right)$ and $\lambda_{3}\left(\omega \right)=0$ and

(9) $$a_{i}={\left(\frac{\mu_{i}}{R_{c}}\right)}^{2}\;\;and\;\;B_{i}=2\frac{{\left(R_{c}/\mu_{i}\right)}^{2}}{\mu_{i}^{2}-1}$$

where $\mu_{i}$ are the roots of $J_{1}^{\prime }\left(\mu_{i}\right)=0$ and $J_{1}^{\prime }\left(.\right)$ is the Bessel function of the first kind and order (Lundell, Nilsson, Dyrby, Parker, Cristinacce, Zhou, Topgaard, Lasič, 2019, Nilsson, Lasič, Drobnjak, Topgaard, Westin, 2017, Stepišnik, 1993) and $R_{c}$ is the cylinder radius.

For a sphere, $\lambda_{1}\left(\omega \right)=\lambda_{2}\left(\omega \right)=\lambda_{3}\left(\omega \right)=\lambda \left(\omega \right)$ and

(10) $$a_{i}={\left(\frac{\mu_{i}}{R_{s}}\right)}^{2}\;\;and\;\;B_{i}=2\frac{{\left(R_{s}/\mu_{i}\right)}^{2}}{\mu_{i}^{2}-2}$$

where $\mu_{i}$ are the roots of the derivatives of the first order spherical Bessel function $j_{1}^{\prime }\left(\mu_{i}\right)=0$ and $R_{s}$ is the sphere radius. $D_{0}$ is fixed at $3\mu m^{2}/ms$ for the sphere, as proposed in (Palombo et al., 2020) and $D_{0}=D_{\text{in}}^{||}$ for cylindrical geometry.

#### 2.1.2 Stick + Ball + Sphere ($R_{\mathrm{cylinder}}=0$) (Original SANDI Model)

We define a three-compartment model, Stick + Ball + Sphere to investigate the sensitivity of the diffusion signal to the sphere radius and signal fraction. This model is the same as the original SANDI model with the difference that here we use b-tensor encoding and frequency-domain analysis.

(11) $$S/S_{0}=f_{\text{stick}}A_{\text{stick}}+f_{\text{sphere}}A_{\text{sphere}}+f_{\text{ball}}A_{\text{ball}}$$

#### 2.1.3 Stick + Ball (Behrens et al., 2003) ($f_{\text{sphere}}=0$ and $R_{\text{cylinder}}=0$)

In this section, we compare the Stick + Ball + Sphere model with a Stick + Ball model and provide the range of sphere signal fractions and radii that make these two models significantly different. This latter model is the simplest model and does not have any time dependency.

(12) $$S/S_{0}=f_{\text{stick}}A_{\text{stick}}+f_{\text{ball}}A_{\text{ball}}$$

## 3 Method

Using both numerical simulations and *in vivo* experiments in healthy volunteers, we explore estimation of the soma size and density $R_{\text{sphere}}$ and $f_{\text{sphere}}$ using the combination of efficient gradient waveforms for LTE, PTE, and STE. Note: as we analyze the powder-averaged/orientationally-averaged signal, we do not need to estimate orientational dispersion. We study the challenges of the fitting landscape, the effect of noise, the lower limit on detectable sphere signal fraction, the empirical lower limit on detectable sphere radius, and the challenge of disentangling two time-dependent properties (cylinder and sphere radius) of the model.

In this work, three models are fitted to the data; cylinder + ball + sphere, stick + ball + sphere, and stick + ball. The number of independent parameters are provided in Table 1.

**Table 1:** The summary of models used in this study as well as the number of parameters.

Model	Parameters	Number of independent parameters
cylinder + ball + sphere	$f_{\mathrm{stick}}$, $f_{\mathrm{ball}}$, $f_{\mathrm{sphere}}$, $D_{\mathrm{in}}^{||}$, $D_{\mathrm{ball}}$, $R_{\mathrm{cylinder}}$, $R_{\mathrm{sphere}}$	6 ($f_{\mathrm{stick}}+f_{\mathrm{ball}}+f_{\mathrm{sphere}}=1$)
stick + ball + sphere	$f_{\mathrm{stick}}$, $f_{\mathrm{ball}}$, $f_{\mathrm{sphere}}$, $D_{\mathrm{in}}^{||}$, $D_{\mathrm{ball}}$, $D_{0}$, $R_{\mathrm{sphere}}$	5 ($f_{\mathrm{stick}}+f_{\mathrm{ball}}+f_{\mathrm{sphere}}=1$
		and $D_{0}=3\mu m^{2}/ms$)
stick + ball	$f_{\mathrm{stick}}$, $f_{\mathrm{ball}}$, $D_{\mathrm{in}}^{||}$, $D_{\mathrm{ball}}$	3 ($f_{\mathrm{stick}}+f_{\mathrm{ball}}=1$)

### 3.1 Noise sensitivity

To explore the sensitivity of parameter estimation to noise perturbations, we simulated three different scenarios: (i) addition of Gaussian noise to the magnitude of the signal; (ii) addition of Gaussian noise to the real and imaginary channels which results in Rician-distributed magnitude signal; and (iii) addressing the noise-floor problem in case (ii) with a (simple) correction. In general, when there is Gaussian noise in the signal, averaging improves the signal to noise ratio (SNR) and because of the orientational-averaging used in this work, we expect some improvement in the SNR in the first scenario and therefore better estimates of the model parameters. In the second scenario, the signal is corrupted by Rician-distributed noise, and therefore the orientational-averaging that improved the SNR in case (i), does not remove the non-zero positive ’noise-floor’ bias in Rician-distributed noise and therefore we expect some bias in the parameter estimates. In the third scenario, we use a simple correction for the Rician bias in case (ii). To estimate the standard deviation of the noise, we include the noise floor in the model so that the predicted signal is $S_{n}=\sqrt{S^{2}+\sigma^{2}}$ where S is our original model prediction and $S_{n}$ is the prediction after accounting for the noise floor (Eichner, Cauley, Cohen-Adad, Möller, Turner, Setsompop, Wald, 2015, Jones, Basser, 2004, Koay, Özarslan, Basser, 2009, Shemesh, 2018). We expect some improvement in the parameter estimates in case (iii) compared to case (ii) but the results of case (i) are expected to be best out of all three cases. We note that for a singlecoil acquisition the magnitude signal is the Rician-distributed envelope of the complex signal (Aja-Fernández, Tristán-Vega, Hoge, 2011, Gudbjartsson, Patz, 1995, Koay, Özarslan, Basser, 2009). With parallel imaging, noise is not Rice-distributed but rather follows a noncentral Chi distribution (Aja-Fernández, Tristán-Vega, 2012, Aja-Fernández, Tristán-Vega, Hoge, 2011, Veraart, Rajan, Peeters, Leemans, Sunaert, Sijbers, 2013) but it behaves similarly in terms of a noise floor.

### 3.2 Lower bound on resolvable sphere signal fraction

Here we considered that the framework had sensitivity to the sphere signal fraction if the inclusion of a sphere component to a stick + ball model was statistically supported by an F-test. To determine the lower bound on the spherical signal fraction and the radius that can be detected using a diffusion-weighted signal, we systematically varied both parameters, while comparing the fit from two models: (i) the stick + sphere + ball model (three-compartments including a spherical component); and (ii) a stick + ball model (two-compartment without a spherical component). To test whether inclusion of the spherical compartment was needed to describe the signal (thereby showing sensitivity to this component) we considered the stick + sphere + ball model justified if the p-value from the F-test was less than 0.05 (Lampinen, Szczepankiewicz, Mårtensson, van Westen, Hansson, Westin, Nilsson, 2020, Nilsson, Alexander, 2012, Panagiotaki, Schneider, Siow, Hall, Lythgoe, Alexander, 2012). Here, the F-statistic is calculated as $F=\left({\text{SSR}}_{1}-{\text{SSR}}_{2}\right)\left(N-M_{2}\right)/\left({\text{SSR}}_{2}\left(M_{2}-M_{1}\right)\right)$ where SSR is the sum of squared residuals, M is the number of fitted parameters of the simplified (1) and full SANDI model (2), and N is the number of measurements. The p-value is estimated using $p=1-\mathrm{fcdf}(F,M_{2}-M_{1},N-M_{2})$ where fcdf is the cumulative distribution function of F-distribution.

### 3.3 Stick + Ball + Sphere *vs* Cylinder + Ball + Sphere

If ultra-strong gradients and diffusion-time settings are such that we do, indeed, have sensitivity to the intra-neurite perpendicular diffusivity, then the intra-neurite compartment should be more correctly modeled using cylinders instead of sticks (Veraart et al., 2020). This introduces an additional challenge, as we now have two compartments (sphere and cylinder) with a diffusion-time dependence. To explore this, we conducted further simulations to investigate the impact of including a non-zero perpendicular intra-neurite diffusivity (or, rather, a cylinder with a finite radius of $R_{\text{cylinder}}=4\;\mu m$) on estimation of sphere radius.

### 3.4 Empirical lower bound on sphere radius

To identify the empirical lower bound on sphere radius, we simulated signals for experiments with fixed diffusion time, Δ=37.05ms, and δ=29.65ms (matching our *in vivo* experimental set-up), diffusivities $D_{\text{in}}^{||}=2\;\mu m^{2}/ms$ and $D_{\text{ball}}=1\;\mu m^{2}/ms$, but with variable sphere signal fractions.

We do not use a fixed step size for all range of $f_{\mathrm{sphere}}$ values, from 0.01 to 0.1 the step size is 0.01, then we have 0.15, and from 0.2 to 1 the step size is 0.1, and therefore we have $f_{\mathrm{sphere}}=\left(0.01,\;0.02,\;0.03,\;...,\;0.1,\;0.15,\;0.2,\;0.3,\;0.4,\;...,\;1\right)$, $f_{\text{ball}}=f_{\text{cylinder}}=\left(1-f_{\text{sphere}}\right)/2$, and sizes, $R_{\text{sphere}}=\left(1,\;1.5,\;2,\;...,\;10\right)\;\mu m$. For each set of signals, we fitted a power-law (McKinnon, Jensen, Glenn, Helpern, 2017, Veraart, Fieremans, Novikov, 2019) to the direction-averaged signal from the LTE measurements for $b=6,7.5,9,10.5ms/\mu m^{2}$ according to ($S/S_{0}=\beta b^{-\alpha }$) and then compared the values of the exponent, α, with values observed empirically *in vivo* to establish a lower bound on the sphere radius. The rationale behind the choice of using the power-law to drive an empirical conclusion is that it is free of any model assumptions, and simply considers the rate of signal decay versus b-value. We know that the α value for a pure stick-like geometry is 0.5, and thus any deviation from this value is indicative of sensitivity to an additional compartment (a deviation from the stick-like geometry could be due to any shape that is not stick-like). The compartment that we choose to change is, in fact, a spherical compartment. By systematically increasing the size of the spherical compartment until such a deviation is detected, we can obtain an empirical lower bound on the spherical compartment. Any sensitivity to the intra-axonal perpendicular diffusivity would make the signal decay faster (Veraart et al., 2020) but with the timing parameters used here, we do not expect any such sensitivity.

### 3.5 Simulations

The numerical simulations were performed using the model in Eq (2), with $f_{\text{sphere}}=0.01:0.01:0.1$, 0.15, 0.2:0.1:0.8, $f_{\text{ball}}=f_{\text{stick}}=\left(1-f_{\text{sphere}}\right)/2$, $D_{\text{in}}^{||}=2\;\mu m^{2}/ms$, $D_{\text{ball}}=0.6\;\mu m^{2}/ms$, $R_{\text{sphere}}=1:0.5:10\;\mu m$, and $R_{\text{cylinder}}=4\;\mu m$. The reason for assuming an equal signal fraction for the ball compartment and the stick compartment is to simplify the simulation. We have also examined a scenario where stick signal fraction is fixed to 0.7 (S1). The simulated protocol matched the *in vivo* protocol and comprised 10 b=0 and 8 non-zero shells ($b=1,2,3,4.5,6,7.5,9,10.5\;ms/\mu m^{2}$) in (10,31,31,31,31,61,61,61,61) directions for LTE and 5 shells ($b=1,2,3,4.5,6\;ms/\mu m^{2}$) in (31,31,31,31,61) directions for PTE and 5 shells for STE ($b=0.2,1,2,3,4.5\;ms/\mu m^{2}$) in (6,9,9,12,15) directions and SNR = 50 with Rician noise. The 61 and 31 directions were optimized based on (Knutsson, 2018). The noisy diffusion signal was modeled according to the following:

(13) $$S_{n}=\sqrt{{\left(S+N_{r}\left(0,\sigma \right)\right)}^{2}+N_{i}{\left(0,\sigma \right)}^{2}}$$

where $S_{n}$ and S are the noisy and noise-free signals, respectively, and $N_{r}$ and $N_{i}$ are the normal distributed noise in the real and imaginary images respectively with a standard deviation of σ (Aja-Fernández, Vegas-Sánchez-Ferrero, Jones, Basser, 2004, Pieciak, Aja-Fernández, Vegas-Sánchez-Ferrero, 2016, Pieciak, Rabanillo-Viloria, Aja-Fernández, 2018, Pieciak, Vegas-Sánchez-Ferrero, Aja-Fernández, 2016). Here SNR level is defined as 1/σ. For each b-tensor shape, and for each b-value, the diffusion signal was averaged over all directions in a shell.

We assumed $D_{\text{ball}}=1\mu m^{2}/ms$, in our power-law experiments (section 3.4), according to an extracellular volume fraction between 5−20% and tortuosity between 1.6-2.1 (Nicholson and Hrabětová, 2017) and the one in section 3.5 is assumed $0.6\mu m^{2}/ms$ to consider more challenging scenarios where there is tissue pathology.

### 3.6 *In vivo* data

Two healthy participants who showed no evidence of a clinical neurologic condition were scanned with the approval of the Cardiff University School of Psychology Ethics Committee. Magnetization-prepared rapid gradient echo (MPRAGE) images were also acquired for anatomical reference. 192 sagittal slices with TE = 2.3 ms, TR = 1900 ms, TI = 900 ms, and a voxel size of 1 mm isotropic and 256×256 matrix size were acquired in 5 minutes.

Diffusion-weighted images were acquired with the protocol detailed in the simulation section 3.5 on a 3T Connectom MR imaging system with 300 mT/m gradients (Siemens Healthineers, Erlangen, Germany). Forty-two axial slices with 3mm isotropic voxel size and a 78×78 matrix size, TE = 88 ms, TR = 3000 ms, partial Fourier factor = 6/8, and heat dissipation limit = 1, were obtained for each individual. The total acquisition time was around one hour. To take full advantage of q-space trajectory imaging, it is imperative to respect the constraints imposed by the hardware, while at the same time maximizing the diffusion encoding strength. Sjolund et al. (Sjölund et al., 2015) provided a tool for achieving this by solving a constrained optimization problem that accommodates constraints on maximum gradient amplitude, slew rate, coil heating, and positioning of radiofrequency pulses. The gradient waveform is optimized and Maxwell-compensated (Szczepankiewicz et al., 2019) based on a framework that maximizes the b-value for a given measurement b-tensor shape and echo time. Substantial gains in terms of reduced echo times and increased signal-to-noise ratio can be achieved, in particular as compared with naive planar and spherical tensor encoding. Duration of the first, pause, and the second waveform in Fig 2 were [29.6, 7.4, 29.6] ms for LTE and [35.6, 7.4, 28.6] ms for PTE and STE. The slew rate was 13.8, 62, and 51.1 mT/m/ms for LTE, PTE, and STE, respectively.

### 3.7 Preprocessing

The diffusion weighted images were corrected for Gibbs ringing (Kellner et al., 2016). We acquire some interleaved b0 images between the diffusion-weighted images (DWIs) to use for motion correction. In PTE and STE data, we registered (linearly) the interleaved b0 images to the first b0 image and used the corresponding transformation to correct the motion in the DWIs. In LTE data the eddy current and subject motion were corrected by FSL EDDY (Andersson and Sotiropoulos, 2016) and finally the gradient nonlinearity was corrected by the method proposed by Rudrapatna et al. (Rudrapatna, Parker, Roberts, Jones, 2018, Rudrapatna, Parker, Jamie, Jones, 2020).

We applied a 3D Gaussian filter with a standard deviation of 0.5 and a full width half maximum of (FWHM) 1.18 to the preprocessed data to make the images smooth. We normalized the direction-averaged signal based on the b = 0 $s/mm^{2}$ signal in each voxel.

### 3.8 Regions of interest

**Fig. 1:**
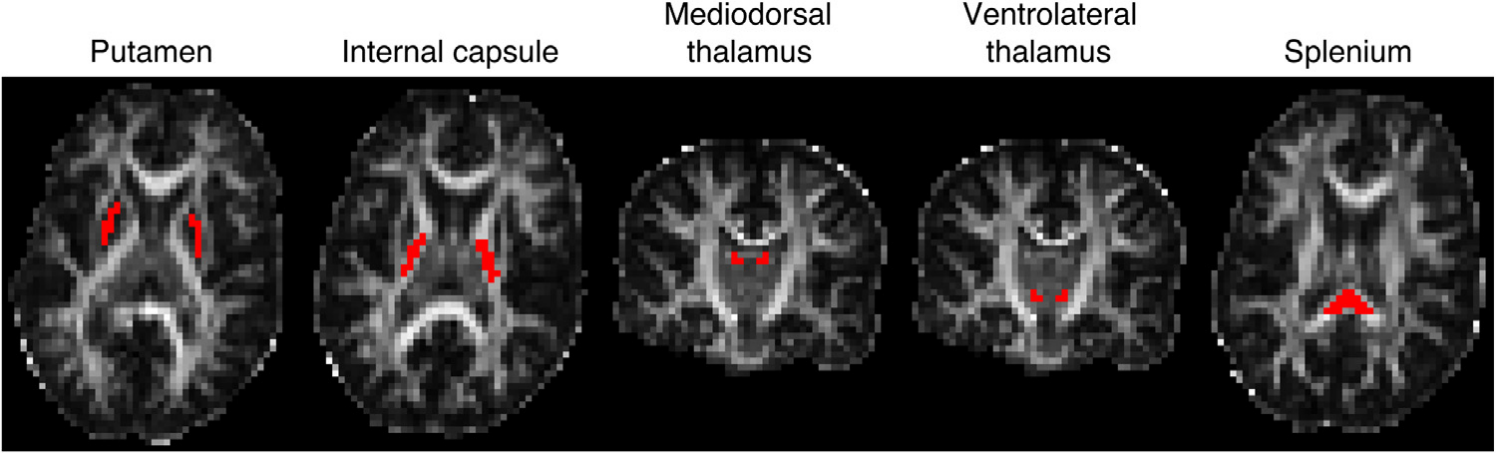
Location of the five ROIs used for the quantitative analysis of this study overlaid on the FA image of one subject. The posterior limb of the internal capsule, splenium, putamen, ventrolateral thalamus, and mediodorsal thalamus are illustrated as red ROIs on the FA map.

We defined five regions of interest (ROIs) in the splenium and internal capsule as white matter regions and putamen, ventrolateral thalamus, and mediodorsal thalamus, as gray matter regions. We selected these regions to study the presence of the spherical compartment in both white matter and gray matter. The internal capsule and splenium were chosen as highly organised regions of white matter brain tissue and putamen, mediodorsal thalamus and ventrolateral thalamus were selected as being gray matter (although thalamus contains some white matter). We selected these as regions to minimize partial volume effects. We did not include cortical gray matter ROI because we have used a 3 mm isotropic voxel size which has some contributions from CSF (i.e., with this resolution we cannot get a pure cortical gray matter voxel). Therefore, we decided to exclude cortical GM from the analysis and focus on GM regions where partial volume with CSF is less problematic, given our voxel size. To define anatomical regions of interest, publically-available atlases were co-registered to each participant’s T_1_-weighted MPRAGE image (affine registration). The white matter ROIs were obtained from the JHU atlas (Mori et al., 2005), while the putamen ROI was obtained from the work of Tziorti et al. (Tziortzi et al., 2011) and ventrolateral thalamus and mediodorsal thalamus from (Danos et al., 2003). We have to mention that because of the relatively low resolution of the DW images (3 mm isotropic), the ROI for putamen may include globus pallidus as well. The MPRAGE image was co-registered to the diffusion-weighted data, and the resulting transform applied to the ROIs to translate them to the native diffusion-weighted space. The five ROIs are illustrated in Fig 1.

### 3.9 Goodness of fit for *in vivo* data

To check the stability of the model fit and that the global minima of the cost function had been found, we first fixed the signal fraction of the spherical compartment and estimated the remaining parameters (The same procedure was used by Lampinen et al. (Lampinen et al., 2019) for finding the stick fraction that can be detected reliably). To assess the precision of parameter estimation, a metric of the goodness-of-fit (see below) was plotted for different values of sphere signal fraction, which was varied systematically between zero and one in 40 equal steps. If the model determined all parameters unequivocally, a clear optimum in the goodness-of-fit would be seen for some sphere signal fractions. Conversely, a flat plot of the goodness-of-fit over a wide range of sphere signal fractions would indicate degeneracy in the fitting, i.e. two or more sets of solutions yield a similarly good fit. For each ROI, all the voxels contained therein were concatenated to provide a sufficient number of data points for our estimation.

Goodness-of-fit was determined using reduced chi-square, $\chi_{\text{red}}^{2}$ (Andrae et al., 2010). The reduced chi-square or normalized residual variance (NRV) (Lampinen et al., 2019) was obtained by dividing the residual variance ($\sigma_{R}^{2}$) by the variance of noise ($\sigma_{\text{noise}}^{2}$):

(14) $$\chi_{\text{red}}^{2}=\sigma_{R}^{2}/\sigma_{\text{noise}}^{2}=\left[\sum n_{i}{\left(S_{i}-S_{i}^{\prime }\right)}^{2}/\left(n-k\right)\right]/\left(\sigma_{\text{noise}}^{2}/n_{\text{voxel}}\right)$$

where $n_{i}$ is the number of directions for ith b and $b_{\Delta }$. $S_{i}$ and $S_{i}^{\prime }$ are the direction-averaged measured and predicted signals, n is the number of samples and k is the number of free parameters in the model. The standard deviation of the noise ($\sigma_{\text{noise}}^{2}$) was estimated for each ROI.

## 4 Results

### 4.1 Simulations

**Fig. 2:**
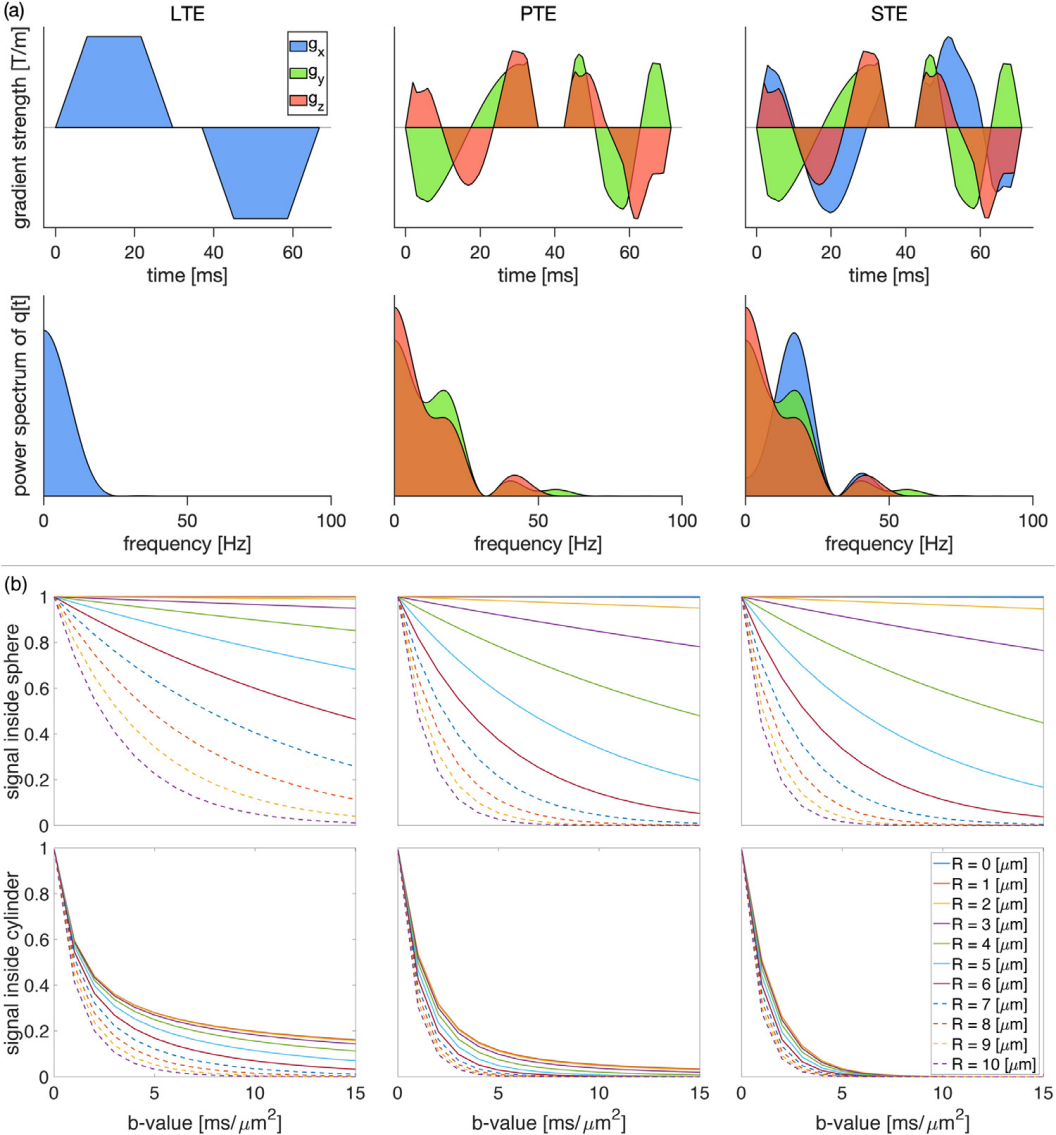
(a) The free gradient waveforms of the linear, planar, spherical tensor encoding and the corresponding frequency power spectra. (b) The signal decay inside the spherical and cylindrical compartments using different encoding schemes and different radii

Fig 2 (a) shows the gradient waveforms used for the linear, planar and spherical tensor encoding and their corresponding frequency spectra. Fig 2 (b) shows the signal decay inside the spherical and cylindrical compartments using different encoding schemes. It is important to note that, for a given b-value, STE results in the most signal loss, followed by PTE and then LTE. LTE appears relatively insensitive for small radii spherical compartments. The results of simulation in Fig 2 (b) show that if we use linear tensor encoding (LTE) and change the b-value from 0 to 15 $ms/\mu m^{2}$, for R=0,1,2μm the signal decay is negligible and therefore insensitive to the sphere radius. When R≥3μm the signal get sensitive to the sphere radius and by increasing the b-value this sensitivity increases. In the case of planar and spherical tensor encoding (PTE and STE), in the domain R≥2μm the signal attenuation becomes sensitive to the sphere radius and by increasing the b-value this sensitivity increases.

#### 4.1.1 Fitting landscape

**Fig. 3:**
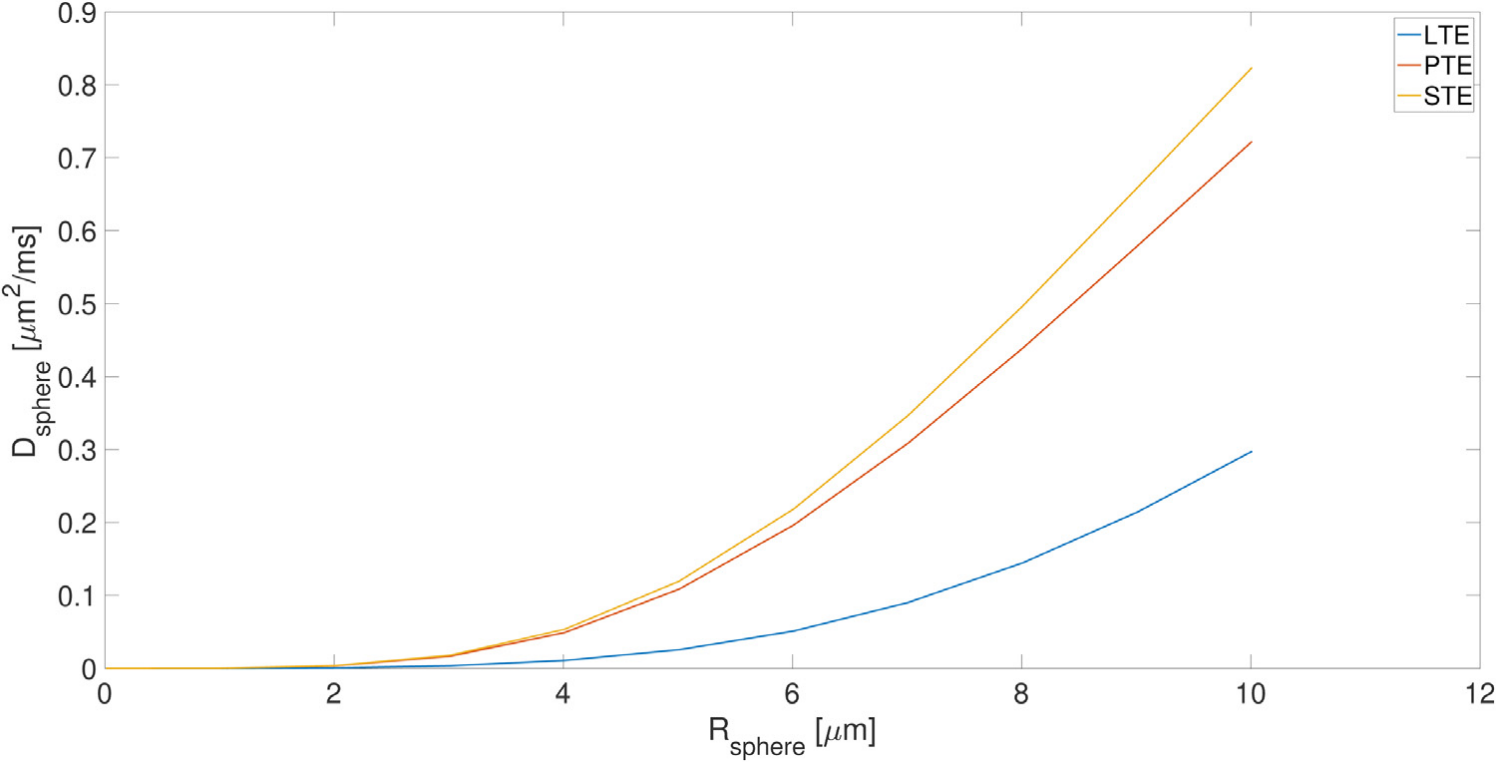
The changes in the apparent diffusivity ($D_{\text{sphere}}$) *versus* the radius of the sphere ($R_{\text{sphere}}$) for linear, planar and spherical tensor encoding (LTE, PTE, and STE)

Fig 3 shows the changes in apparent diffusivity of the spherical compartment ($D_{\text{sphere}}$) as a function of radius of the spherical compartment for the three b-tensor shapes. Clearly, there is a large difference in the sensitivity to sphere radius, with LTE being the least sensitive and PTE and STE tracking each other closely in the plot of $D_{\text{sphere}}$ *vs* $R_{\text{sphere}}$. Notably, for all wave-forms, there is little differentiation in sphere signal attenuation for low radii, (e.g. $R_{\text{sphere}}<4\mu m$).

#### 4.1.2 Noise sensitivity

**Fig. 4:**
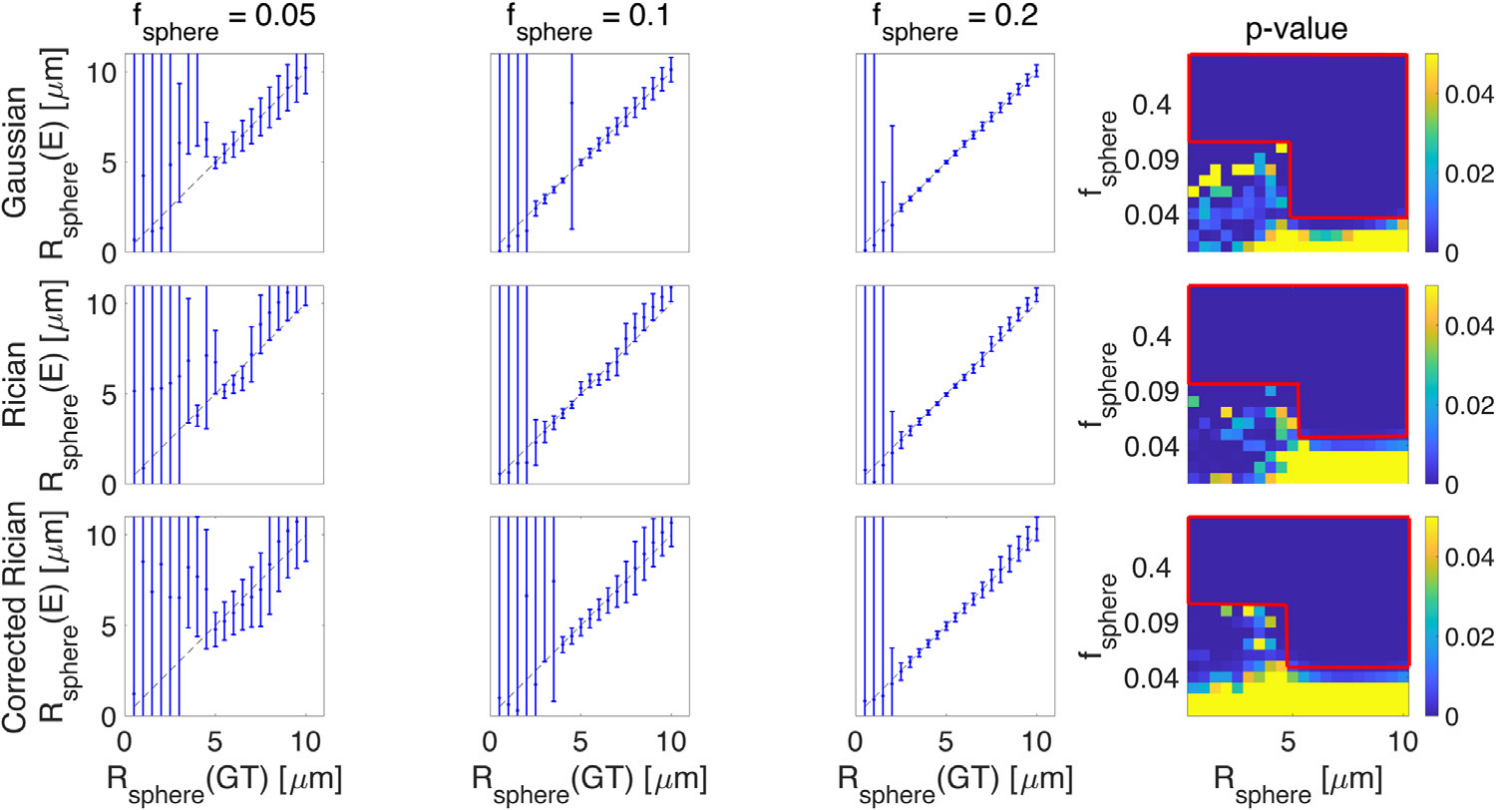
The results of fitting (stick + ball + sphere) the sphere radius for different sphere signal fractions (GT = Ground Truth and E = Estimated). The figure also shows the p-value of the F-test in the presence of Gaussian, Rician, and corrected Rician noise respectively. The red rectangles in the right side plots show the areas that the three-compartment model is significantly different from the two-compartment model (three-compartment model (stick + ball + sphere) is preferred over the two-compartment model (stick + ball)). The diagonal black line is the line of identity and the error bars show the confidence interval.

Fig 4 shows the results of fitting the sphere radius (stick + ball + sphere) for different sphere signal fractions under different noise simulations. The figure also shows the p-value of the F-test between the two and three-compartment models in the presence of Gaussian, Rician, and corrected Rician noise. Here, we take p<0.05 as an indication that the full model (three-compartment) is preferred over the simplified model (two-compartment). When the sphere radius or the signal fraction of the sphere is small ($R_{\text{sphere}}<2\mu m$ and $f_{\text{sphere}}<0.05$) the simplified model is preferred. Fig 4 shows that the estimates are largely positively biased in the Rician-noise data, whereas their errors are symmetrically-distributed about the line of identity in Gaussian only. After Rician noise correction, it looks more like the Gaussian - in terms of symmetrical distribution around the line of identity.

For small radii, the noise dominates over measurable effects. The error bars in Fig 4 show the confidence interval. In the fitting, we use *lsqcurvefit* in MATLAB which returns the jacobian at the solution, and then *nlparci* command is used to find the 95% confidence interval.

#### 4.1.3 Sphere signal fraction resolution limit

The examination of the F-test measures indicates a lower bound on the sphere signal fraction that can be detected or reasonably modeled from the diffusion-weighted signal. This is around 10% for SNR = 50 (Fig 4) but this limit changes at different noise levels (Fig S5). The figure shows that for very small sphere radius or very low sphere signal fraction, the ball + stick model is preferred. With the protocol used in this study, below 3μm there is not any sensitivity to the spherical compartment, while above 6μm there is definitely sensitivity to the spherical compartment and between 3 to 6μm, we can optimize the sequence to make the signal sensitive to the spherical compartment (based on Fig. 3 and 4). The changes of sphere radius estimates versus ground truth for a wider range of SNR values are shown in this link: https://bit.ly/SphStickBall. The lower limit scales inversely with SNR, i.e. as SNR increases, smaller sizes are detectable.

#### 4.1.4 Stick + Ball + Sphere *vs* Cylinder + Ball + Sphere

**Fig. 5:**
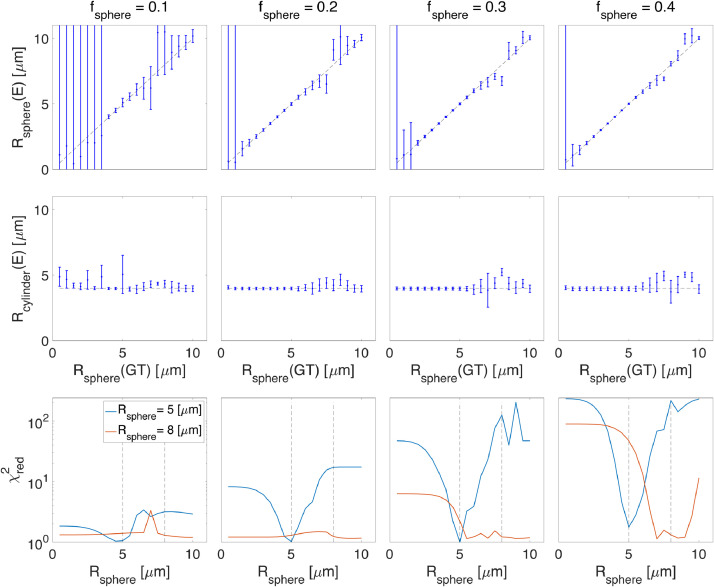
Estimated sphere and cylinder radii versus the ground truth sphere radius values for cylinder + ball + sphere model (SNR=200). The third row shows the reduced chi-square, $\chi_{\text{red}}^{2}$, values for two scenarios where the sphere radius is 5 and 8 μm, blue and red curves respectively We take the simulated signal generated for $R_{\mathrm{sphere}}=5$ and instead of estimating all the parameters of the model which are $f_{\mathrm{cylinder}}$, $f_{\mathrm{ball}}$, $f_{\mathrm{sphere}}$, $D_{\mathrm{in}}^{||}$, $D_{\mathrm{ball}}$, $R_{\mathrm{sphere}}$, and $R_{\mathrm{cylinder}}$, we fix $R_{\mathrm{sphere}}$ to 0.5,1,1.5,...,10μm and estimate the remaining parameters and plot the reduced chi-square as a function of $R_{\mathrm{sphere}}$ (blue curve). If there is not degeneracy, we expect to see a sharp minimum in the $\chi_{\text{red}}^{2}$ curve at $R_{\mathrm{sphere}}=5$ which is clear in the figure. We repeat the same strategy for $R_{\mathrm{sphere}}=8\mu m$ (the red curve) where the $\chi_{\text{red}}^{2}$ curve has several local minima or even flat which shows the presence of degeneracy when $R_{\mathrm{sphere}}$ is larger than 5μm. This behavior can be explained by the swap of time dependent parameters ($R_{\mathrm{sphere}}$ and $R_{\mathrm{cylinder}}$) in the fitting which leads to unstable fitting of the model parameters. (GT = ground truth and E = estimated).

In addition to the challenge of estimating sphere size (given the above 3 points), the fitting becomes more challenging if we have cylinders instead of sticks because in this model the time-dependence of the signal can now arise from two independent sources (the cylinder and the sphere). Fig 5 shows the estimated sphere size when there is a non-zero diameter cylinder, (SNR = 200). For sphere signal fractions smaller than 0.2 the estimated sizes deviate from the ground truth. At high radii the signal decay of the spherical compartment can be similar to the signal decay of the cylinder compartment, making it difficult to uniquely assign signal attenuation, and therefore the estimations for the sphere radius get unstable.

The second row of Fig 5 shows the estimated cylinder radius versus different sphere radii. The ground truth cylinder radius ($R_{\mathrm{cylinder}}=4\mu m$) is presented using the dashed black line. For $f_{\mathrm{sphere}}=0.1$, the estimated cylinder radii deviate from the ground truth (4μm). When increasing the sphere signal fraction ($f_{\mathrm{sphere}}=0.2,\;0.3,\;0.4$), the estimate of cylinder radii becomes unstable for sphere radii larger than 5μm. In the third row we take the simulated signal generated for $R_{\mathrm{sphere}}=5$ and instead of estimating all the parameters of the model which are $f_{\mathrm{cylinder}}$, $f_{\mathrm{ball}}$, $f_{\mathrm{sphere}}$, $D_{\mathrm{in}}^{||}$, $D_{\mathrm{ball}}$, $R_{\mathrm{sphere}}$, and $R_{\mathrm{cylinder}}$, we fix $R_{\mathrm{sphere}}$ to 0.5,1,1.5,...,10μm and estimate the remaining parameters and plot the reduced chi-square as a function of $R_{\mathrm{sphere}}$ (blue curve). If there is no degeneracy, we expect to see a sharp minimum in the $\chi_{\text{red}}^{2}$ curve at $R_{\mathrm{sphere}}=5$ which is clear in the figure. We repeat the same strategy for $R_{\mathrm{sphere}}=8\mu m$ (the red curve) where the $\chi_{\text{red}}^{2}$ curve shows several local minima or a flat curve which represents degeneracy when $R_{\mathrm{sphere}}$ is larger than 5μm. This behavior can be explained by the sharing of time dependent variance between ($R_{\mathrm{sphere}}$ and $R_{\mathrm{cylinder}}$) in the fitting which leads to unstable fitting of the model parameters.

This behavior is observed where the noise floor is very low (SNR = 200) in the simulated signal. The deviation from the ground truth in the presence of noise, for different noise levels, is shown in Fig S6 (https://bit.ly/SphCylBall).

#### 4.1.5 Empirical lower limit on sphere radius

**Fig. 6:**
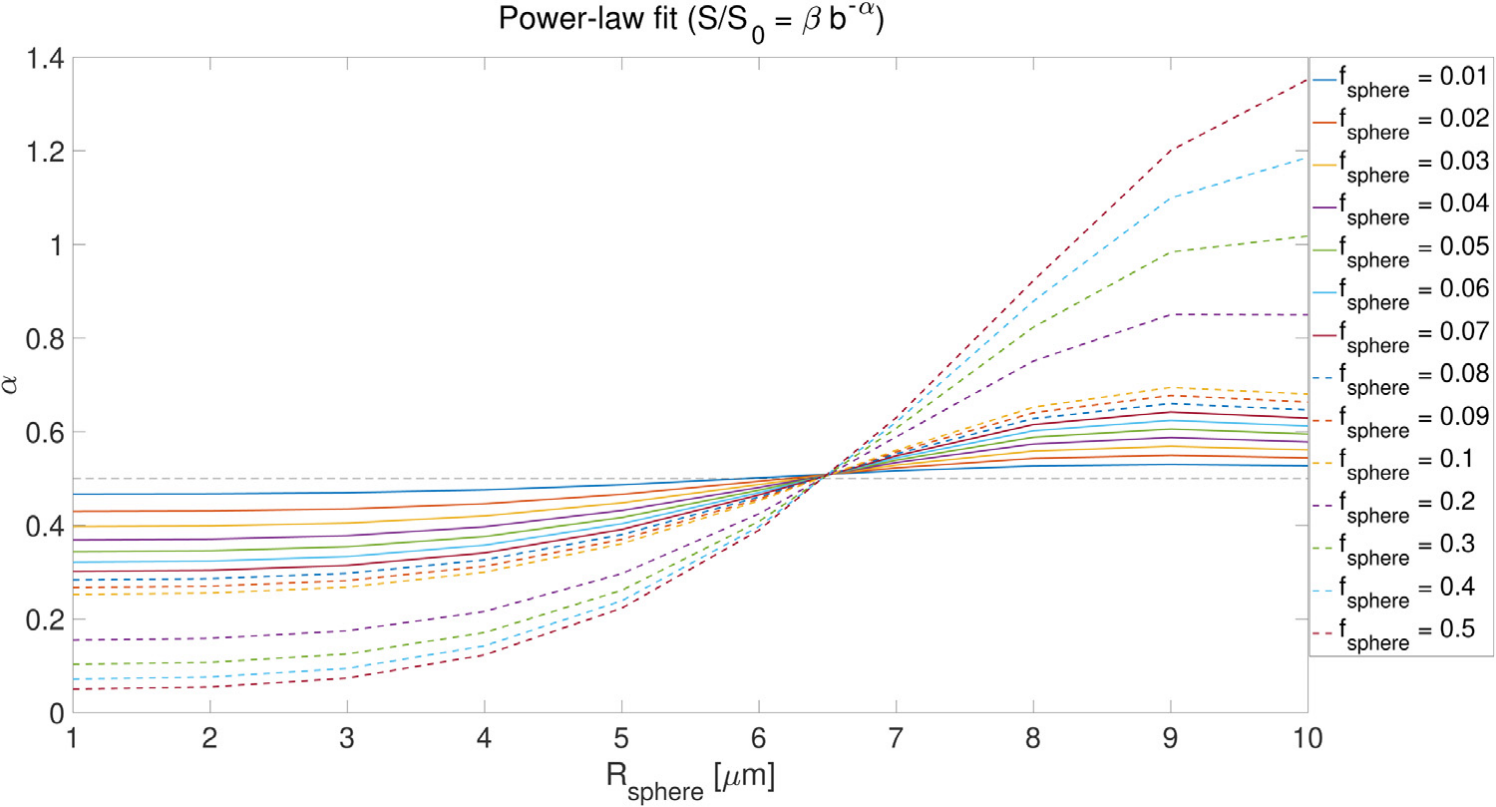
The effect of sphere size and signal fraction on exponent α (similar to Fig. 2 in (Palombo et al., 2018a)). ($f_{\text{sphere}}=0.01:0.01:0.1,\;0.2:0.1:0.5$, $f_{\text{ball}}=f_{\text{stick}}=\left(1-f_{\text{sphere}}\right)/2$, $D_{\text{in}}^{||}=2\;\mu m^{2}/ms$, $D_{\text{ball}}=1\;\mu m^{2}/ms$, $R_{\text{sphere}}=1:1:10\;\mu m$, δ=29.65ms, and Δ=37.05ms).

In addition to the lower bound imposed by the noise floor on the sphere signal fraction and size, there is an empirical lower limit on the sphere radius which can be derived from power-law measurements. Fig 6 shows the effect of sphere signal fraction and size on the estimated exponent, α, in the power-law fit ($S/S_{0}=\beta b^{-\alpha }$) experiments. As will be discussed below, in our *in vivo* data, we observe power-law exponents greater than 0.5 in the white matter. Fig 6 shows that to observe such values of α the sphere radius needs to be larger than 7 microns. Of specific interest for WM, by fixing the stick signal fraction to $f_{stick}=0.7$ and varying the sphere signal fraction, we observe that the sphere resolution limit does not change (Fig S3). Changing $D_{0}$ in the spherical compartment will change the simulation results slightly. For example, decreasing $D_{0}$ to $2\mu m^{2}/ms$ changes the lower bound that is reported based on Fig 6 to 6μm. The contribution from the ball compartment with $D_{\text{ball}}=1\mu m^{2}/ms$ disappears in high b-values. We will discuss the implications of this key result in the context of the *in vivo* data below.

**Fig. 7:**
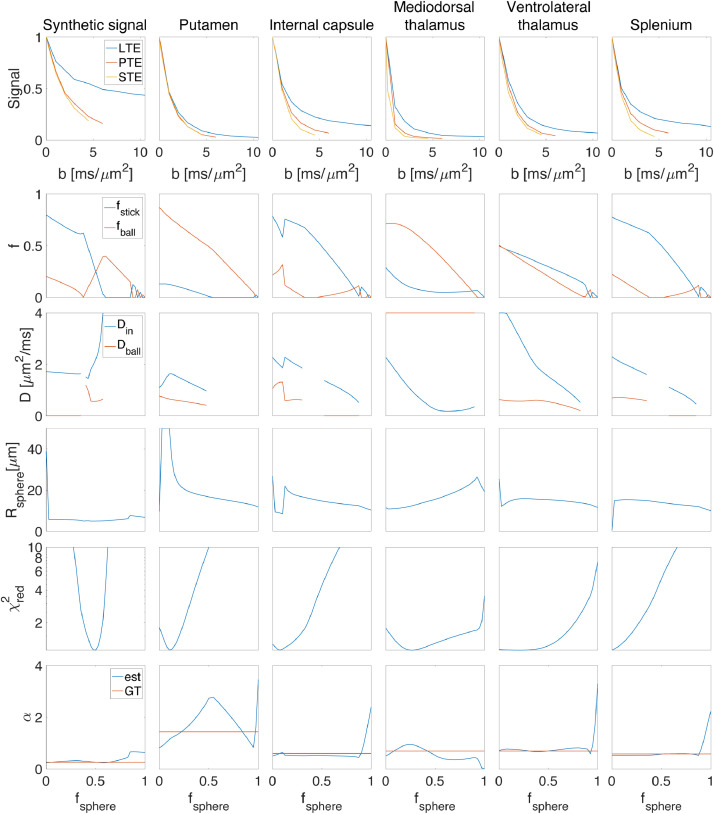
The results of fitting the stick + ball + sphere model to the diffusion-weighted signal by fixing the sphere signal fraction to different values. Five different ROIs of the brain are used here; putamen, internal capsule, mediodorsal thalamus, ventrolateral thalamus, and splenium. The mean value of the direction-averaged signal for each ROI is represented in the first row (in different columns). The second row shows the estimated signal fraction of stick ($f_{\text{stick}}$) and ball ($f_{\text{ball}}$) for different predefined sphere signal fractions (We used f as y-label here to show the signal fraction of both ball and stick in one plot). The third row illustrates the parallel diffusivity of the stick ($D_{\text{in}}^{||}$) and the diffusivity of the ball ($D_{\text{ball}}$) for different ROIs. The estimated radius of the sphere is illustrated in the fourth row. And finally, the last two rows show how well this model can explain the signal for different predefined sphere signal fractions ($f_{\text{sphere}}$) in terms of reduced chi-square and power-law. The first column in the figure shows the results of fitting a synthetic signal generated with the following parameters; $f_{\text{sphere}}=0.5$, $f_{\text{ball}}=f_{\text{stick}}=0.25$, $D_{\text{in}}^{||}=2\;\mu m^{2}/ms$, $D_{\text{ball}}=0.6\;\mu m^{2}/ms$, $R_{\text{sphere}}=5\;\mu m$, and SNR = 100, Rician distributed signal. Note that we do not estimate diffusivity of the compartment when the signal fraction is estimated as zero, this is the reason for discontinuity in the plots of estimated diffusivities.

A good ’sanity check’ is that after reconstructing the signal from the estimated parameters the exponent α in the power-law fit ($S/S_{0}=\beta b^{-\alpha }$) of the original and the modeled signal should not change considerably (see the last row of Fig 7), otherwise, the parameters are representing the signal incorrectly. This approach has several limitations that should be acknowledged; The first limitation is that we used a pre-determined and fixed set of parameters in the simulation. If we had used a smaller diffusivity for the extra-cellular compartment, for the range of b-values used here ($6\leq b\leq 10.5\;ms/\mu m^{2}$), there might have been some residual contribution from the extracellular compartment and therefore the exponent α and the behaviour of the signal would change. The second limitation is that we fixed the intra-neurite and extracellular signal fractions to be equal which may not reflect reality. Despite these limitations, we consider the result a useful empirical benchmark because the fixed diffusivities used in this experiment are close to the range of diffusivities estimated in previous works (Dhital et al., 2019). As such, the contribution from the extracellular compartment at high b-values will be negligible meaning that the only remaining signal contributions come from spheres and sticks.

### 4.2 *In vivo* results

Here we provide *in vivo* results from fitting the Stick + Ball + Sphere model. Fig 7 shows the results of fitting the model to the signal by fixing the sphere signal fraction to different values. Results are shown for five different ROIs: Splenium; internal capsule; mediodorsal thalamus; ventrolateral thalamus; and putamen. Note that, for the fitting, the data points from all three voxels in the ROI are concatenated to provide enough data points for a stable fit, but in the figure, the average of these three voxels is shown. The flat valleys in $\chi_{\text{red}}^{2}$ correspond to plausible sphere signal fractions in different ROIs of the brain, for example, 0-0.125 for splenium and internal capsule and 0.2-0.3 for the putamen (Fig 7). When the valley is flat for a large range of sphere signal fractions, it means the data do not provide a unique solution and a range of parameters can represent the signal equally well. This may be related to a large range of acceptable parameters as shown by the second to the fourth row of Fig 7. For example, acceptable $D_{\text{in}}^{||}$ values for the stick compartment ranged between 2.2 and 2.5 $\mu m^{2}/ms$ in the splenium and between 1.2 and 1.5 $\mu m^{2}/ms$ in the putamen. If there was no degeneracy in the estimation of parameters and the data could provide useful information about the underlying microstructure, then the plot would have a sharp valley at the local optimum. Among the five ROIs we selected in this experiment, we see a quite sharp minimum for the putamen and mediodorsal thalamus (with $f_{\text{sphere}}$ around 0.2-0.3), and the minima for white matter (splenium and internal capsule) clearly puts the sphere fraction in the sub 10% regime, which is where it is expected. The first column of Fig 7 shows the parameter estimates for a synthetic signal generated with the following parameters; $f_{\text{sphere}}=0.5$, $f_{\text{ball}}=f_{\text{stick}}=0.25$, $D_{\text{in}}^{||}=2\;\mu m^{2}/ms$, $D_{\text{ball}}=0.6\;\mu m^{2}/ms$, $R_{\text{sphere}}=5\;\mu m$, and SNR = 100, Rician distributed signal. There is a sharp minimum in $\chi_{\text{red}}^{2}$ which shows there is only one set of parameters that fits the signal accurately. Note that we do not estimate the diffusivity of the compartment when its contribution to the signal (i.e., the signal fraction) is estimated to be zero, which explains the discontinuity in the plots of estimated diffusivities.

**Fig. 8:**
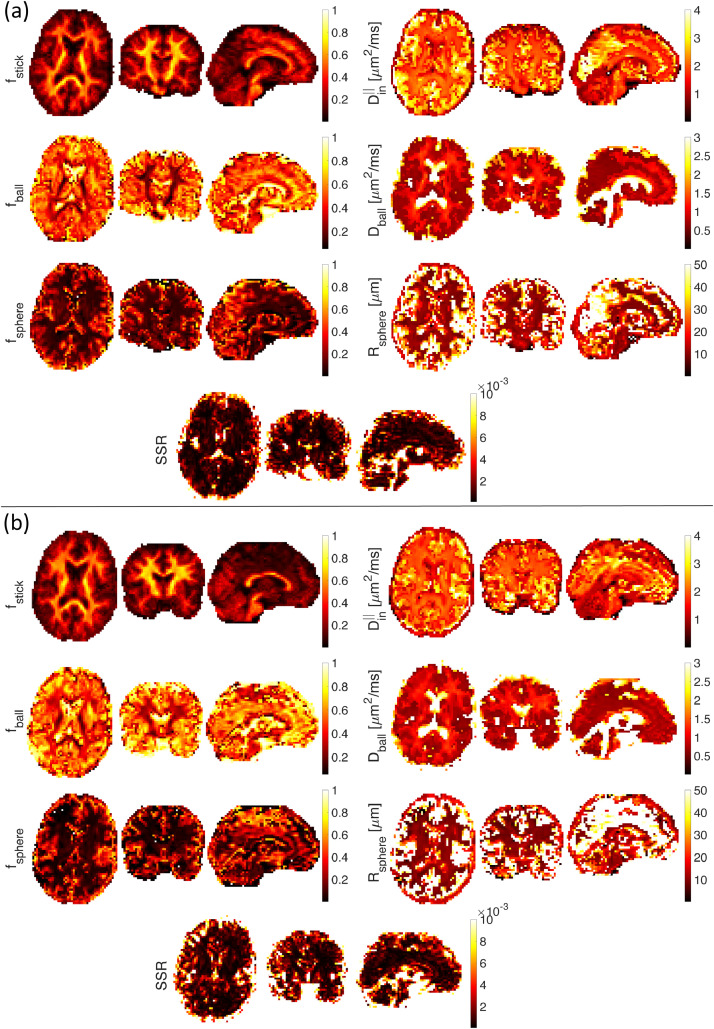
Ball+stick+sphere model without estimation of noise parameter. Estimated stick ($f_{\text{stick}}$), ball ($f_{\text{ball}}$), and sphere ($f_{\text{sphere}}$) signal fractions, intra-axonal parallel diffusivity ($D_{\text{in}}^{||}\left(\mu m^{2}/ms\right)$), extra-cellular diffusivity ($D_{\text{ball}}\left(\mu m^{2}/ms\right)$), and sphere radius ($R_{\text{sphere}}\left(\mu m\right)$) on axial, sagittal, and coronal views of the smoothed brain image ((a) first subject and (b) the second subject) (A 3D Gaussian kernel with standard deviation of 0.5 is used for smoothing).

Fig 8 shows estimated parameter maps *in vivo*, (a) for the first subject and (b) for the second subject. SSE illustrates the sum of squared differences. When fitting on a voxel by voxel-level, the fitting is unstable and the resulting maps are not smooth. Besides, the large voxel size (3 mm) used here and the resulting problems with partial volume may affect the estimated parameters as well. Maps of the estimated parameters in Fig 8 show a reasonable contrast that matches the results in (Palombo et al., 2020). The $f_{\text{stick}}$ map has higher values in white matter tracts in the brain while $f_{\text{sphere}}$ values are higher in the gray matter. The $f_{\text{stick}}$ values in cortical GM range from 0.1 to 0.2 which is in agreement with recent works on estimating neurite density in GM using b-tensor encoding (Lampinen, Szczepankiewicz, Mårtensson, van Westen, Sundgren, Nilsson, 2017, Lampinen, Szczepankiewicz, Novén, van Westen, Hansson, Englund, Mårtensson, Westin, Nilsson, 2019).

**Fig. 9:**
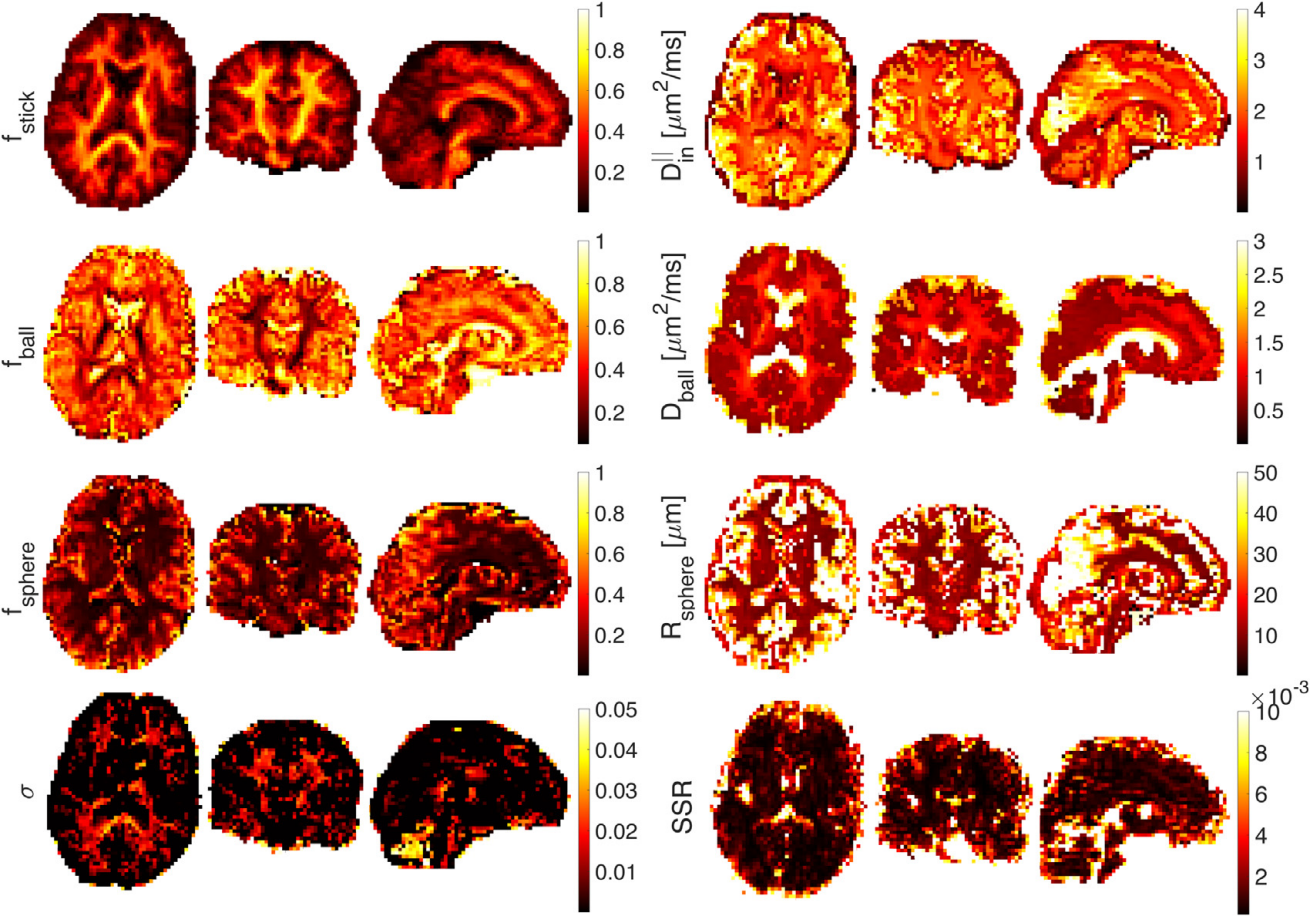
Ball+stick+sphere model with estimation of the noise parameter. Estimated stick ($f_{\text{stick}}$), ball ($f_{\text{ball}}$), and sphere ($f_{\text{sphere}}$) signal fractions, intra-axonal parallel diffusivity ($D_{\text{in}}^{||}\left(\mu m^{2}/ms\right)$), extra-cellular diffusivity ($D_{\text{ball}}\left(\mu m^{2}/ms\right)$), sphere radius ($R_{\text{sphere}}\left(\mu m\right)$), and standard deviation of the noise (σ) on axial, sagittal, and coronal views of the smoothed brain image (A 3D Gaussian kernel with standard deviation of 0.5 is used for smoothing).

Fig 9 illustrates the estimated parameters of the ball+stick+sphere model including the noise floor as an extra parameter to fit. Most of the signal values in the white matter are above the noise floor, so there is not much information conveyed about the level of noise and therefore the estimation of σ values in white matter voxels is challenging.

**Fig. 10:**
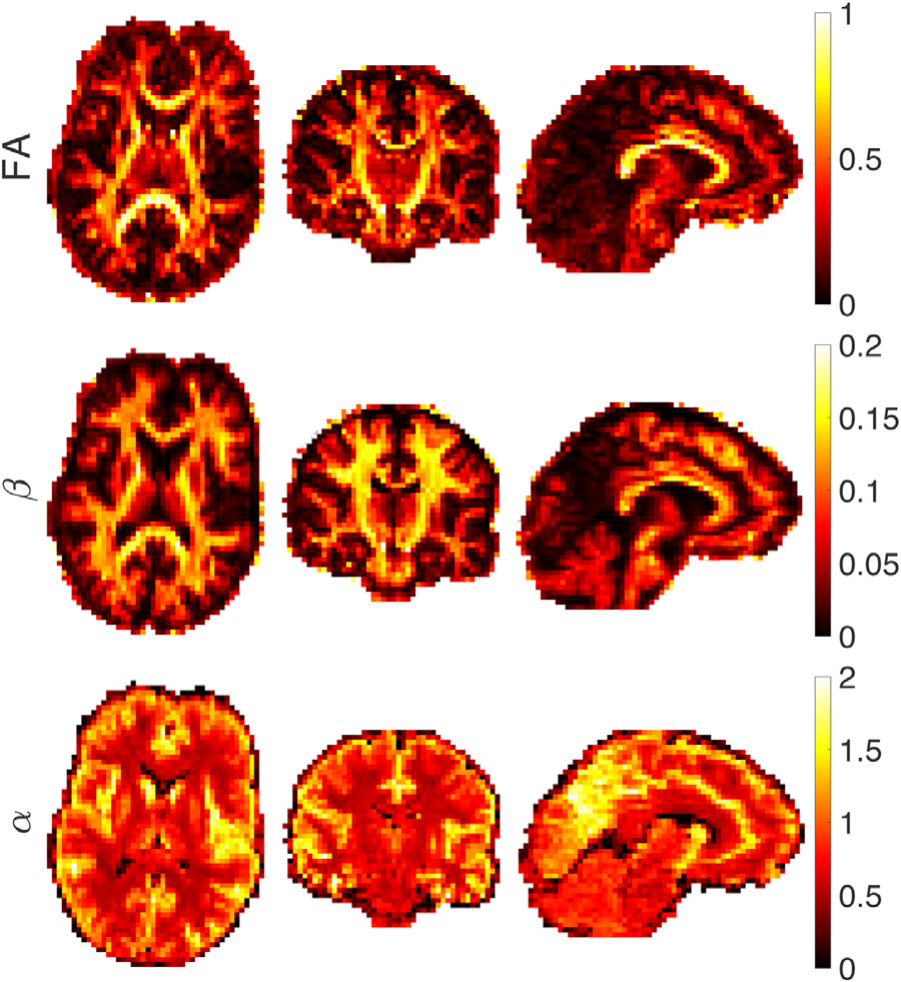
Estimated FA, parameter β and α of the power-law fit ($S/S_{0}=\beta b^{-\alpha }$) from axial, coronal, and sagittal views of the brain image.

Fig 10 shows the results of fitting a power-law ($S/S_{0}=\beta b^{-\alpha }$) to the diffusion-weighted signal. CSF has the fastest decay and therefore no signal remains from CSF in the high b-value data and the α value is close to zero. The decay in the GM is faster than the WM and therefore the estimated α values are correspondingly larger in GM than WM. Within WM, α is usually larger than 0.5. As noted by Veraart et al. (Veraart et al., 2019) and McKinnon et al. (McKinnon et al., 2017), if there is only a stick compartment, then α = 0.5. The α value larger than 0.5 which is associated with a faster decay, may come from the exchange between compartments (Stanisz et al., 1997), sensitivity to the axon diameter (Veraart et al., 2020) or the presence of a non-negligibly-sized third compartment (Palombo et al., 2020) that makes the signal decay faster than α=0.5. If the radius of the spherical compartment is less than approximately 7 microns then the exponent is smaller than 0.5 (α<0.5). As will be seen in the map of α in Fig 10, we do not observe values of α less than 0.5 in the white matter. This places a lower bound on the sphere radii in white matter of around 7 μm (Fig 6). This finding is compatible with what we obtained from F-test in Fig 4.

## 5 Discussion

The SANDI model (Palombo et al., 2020) extends existing multi-compartment models that only consider two pools of water in brain tissue (Alexander, Dyrby, Nilsson, Zhang, 2019, Fieremans, Jensen, Helpern, 2011, Jespersen, Kroenke, Østergaard, Ackerman, Yablonskiy, 2007, Kaden, Kruggel, Alexander, 2016, Novikov, Veraart, Jelescu, Fieremans, 2018, Novikov, Fieremans, Jespersen, Kiselev, 2019, Zhang, Schneider, Wheeler-Kingshott, Alexander, 2012). Palombo et al. (Palombo et al., 2018a) suggested that the failure of the stick model in gray matter (McKinnon, Jensen, Glenn, Helpern, 2017, Veraart, Fieremans, Novikov, 2019) can be due to the abundance of cell bodies (namely soma). In previous works, the contribution from soma was considered as part of the extracellular space (Jespersen, 2012, Jespersen, Kroenke, Østergaard, Ackerman, Yablonskiy, 2007) because the exchange between the restricted water in soma and the hindered water in the extracellular space was assumed to be fast. However, recent studies (Yang et al., 2018a) suggest that the pre-exchange time of intracellular water in neurons and astrocytes is   500ms. These findings prompt the conclusion that for diffusion times much smaller than 500ms (e.g.   10-20 ms) the exchange between intra and extra-cellular water may be negligible, supporting the idea that the signal from spins restricted in soma may be non-negligible.

We caution against drawing comparisons with other models of microstructure that model less information, such as the family of two-compartment microstructural models, including NODDI and the Standard Model. While data for these more simplistic models can be collected in a more clinically-feasible protocol, the acquisition protocol for the current study was around an hour. Developments in MRI hardware and pulse sequences will inevitably shorten the protocol duration in the future, but for now the SANDI protocol remains in the domain of research applications. Continuous advances in image hardware, sequences and reconstruction mean that acquisitions times are getting shorter and shorter, which will allow this extended SANDI protocol to be incorporated into clinical studies. Although the protocol used in this study is too long for clinical applications, we could potentially optimize the acquisition toward clinical studies in the future, e.g. as proposed in (Alexander, 2008, Lampinen, Szczepankiewicz, Mårtensson, van Westen, Hansson, Westin, Nilsson, 2020).

In this work, we studied the minimal sphere signal fraction and radius that can be detected from diffusion MRI of water inside spheres. We performed additional simulations with model parameters more compatible with WM, and we did not observe considerable differences in the results, see Supplementary Information Fig S1. Our finding is in agreement with the results reported by Dhital et al. (Dhital et al., 2018) (2% of the unweighted signal for moderate diffusion times using Prisma scanner) and Tax et al. (Tax et al., 2020) (isotropic signal fraction of 9.7% for the apparent diffusivity of $0.12\mu m^{2}/ms$). It should be noted that the protocol in this study is not specifically optimized for size estimation. For example, intentionally varying the frequency spectra (e.g. to include high frequency components), might result in better sensitivity to smaller pore-sizes (Drobnjak et al., 2013).

The stick signal fraction estimated in the earlier work of Lampinen et al. (Lampinen et al., 2019) did not align with estimates obtained in the current study. However, the estimates from a later study from Lampinen et al. (Lampinen et al., 2020) align more closely with our estimates, presumably because the acquisition sequence was optimized. Comparing the estimation of sphere signal fraction in this work with the stick signal fraction in (Lampinen, Szczepankiewicz, Mårtensson, van Westen, Hansson, Westin, Nilsson, 2020, Lampinen, Szczepankiewicz, Novén, van Westen, Hansson, Englund, Mårtensson, Westin, Nilsson, 2019) we conclude that to estimate the parameters accurately, the acquisition should be optimized toward the estimation of the model parameters.

Here we used a combination of linear, planar, and spherical tensor encoding to ameliorate the degeneracy problem that exists in the fitting of multi-compartment models. Nilsson et al. (Nilsson et al., 2017) reported that for the estimation of diameter in complete orientation dispersion (which we effect by powder-averaging the signal), from an SNR perspective it is advantageous to use oscillating gradient spin echo (OGSE) compared to standard SDE. The benefits of double diffusion encoding (DDE) for size estimation have been presented in other studies (Benjamini, Komlosh, Basser, Nevo, 2014, Katz, Nevo, 2014, Vincent, Palombo, Valette, 2020). Our results suggest using multiple waveforms provides the best estimates.

In the case of cylinder diameter estimation, the resolution limit is determined by the amount of attenuation due to radial diffusion. This attenuation is estimated by the integral of the gradient squared and can be maximized by either a fat-pulse SDE or a rectangular oscillating pulse. However, when the long axis of the cylinder is not perpendicular to the direction of the applied gradient, the high b-values should be avoided because of the signal attenuation and decrease in SNR. To improve the SNR and the resolution limit for cylinder diameter estimation, waveforms should have more oscillations and hence lower bvalues (Nilsson et al., 2017). Here, we are targeting the sphere diameter, and therefore OGSE or SDE can be both useful.

In the estimation of sphere radius from the diffusion-weighted signal, different confounding factors have to be considered. One of the challenges is that for small sphere radii (<3μm), the fitting landscape is flat and there is a negligible change in the signal for small sphere sizes (Fig 3). When there is low sensitivity to some parameters, the numerical optimization algorithm terminates prematurely and therefore the estimates are not accurate. Noise is another confounding factor that affects the estimation of parameters in both model-based and signal representation based techniques. Parameters obtained from multi-compartment models, (the stick + ball + sphere model in this paper) applied to noisy data are biased because of the effect of noise. Three different noise scenarios were simulated here: Gaussian noise, Rician noise, and corrected Rician noise. If the data were corrupted with purely Gaussian noise, then this could be removed to some extent through the act of computing the orientationally-averaged signal. However, as we invariably use the magnitude-reconstructed data, the noise has a Rician distribution, which presents a more challenging scenario because averaging does not remove the bias. This effect is more pronounced when there is a small contribution from the spherical compartment. Using data from high b values only would indeed simplify the model by suppressing extracellular signal and improve fit stability and accuracy, as shown by Fig S7.

We wish to stress that the challenges we identified are mainly relevant to WM. The SANDI model was developed mainly for soma imaging in GM. Nothing in our results suggests that SANDI is unreliable in GM, and will indeed, benefit from the multi-waveform and frequency-domain approach presented here. The challenge in the gray matter is to determine whether the deviation from the ’stick’ model comes from the soma compartment, exchange between compartments (Jelescu and Novikov, 2020) or both.

We note that the extracellular signal fraction obtained from the fit in the gray matter areas is around 0.45 which is higher than expected. This discrepancy can be explained by three factors: first, $T_{2}$ relaxation is not explicitly accounted for in our model, and second, we consider the same proton density in both intra-axonal and extra-axonal spaces. And also non-negligible partial volume with CSF, particularly problematic for cortical GM at $3\;mm^{3}$ resolution. The model presented here is only sensitive to relative signal amplitudes while differences in $T_{2}$ relaxation can impose different weights to the amplitudes of the pools (Callaghan, 1995, Szafer, Zhong, Gore, 1995). The specific assignment of nerve water $T_{2}$ components by simultaneously considering compartmental diffusion and transverse relaxation properties was already studied by Peled et al. (Peled et al., 1999) in myelinated nerves of the frog sciatic nerve tissue. More and more evidence is given for the $T_{2}$ relaxation time constants of intra- and extra-axonal water to be different from each other in case of slow exchange between the intra-axonal and extra-axonal pools (Dortch, Apker, Valentine, Lai, Does, 2010, Peled, Cory, Raymond, Kirschner, Jolesz, 1999). In the case of fast exchange (Szafer et al., 1995), the signal loss in all the pools would be weighted in the same manner. Although it is relatively easy to incorporate relaxation into the model and fit the experimental data with additional parameters (relaxation rates), such a model results in an unstable fit with the current protocol. To incorporate additional parameters (i.e., relaxation rates) in the model, we need to obtain additional information in our experiment which can be achieved by extending the model with compartmental $T_{2}$ relaxation and complementing the protocol with relevant acquisition (Gong, Tong, He, Sun, Zhong, Zhang, 2020, Lampinen, Szczepankiewicz, Mårtensson, van Westen, Hansson, Westin, Nilsson, 2020, Veraart, Novikov, Fieremans, 2018). However, playing out the complex waveforms takes time - and therefore it is challenging to achieve sufficiently short echo times to resolve different compartments (particularly those with short $T_{2}$s) - and the results may be biased towards the compartments with longer $T_{2}$s.

We highlight limitations of the current study in the estimation of spherical compartment signal fraction and size for white matter and gray matter.

Limitations for the white matter; first, we assume that the intra-axonal water comes from straight axons, which is not the case in most of the white matter voxels (Jeurissen, Leemans, Tournier, Jones, Sijbers, 2013, Lee, Papaioannou, Kim, Novikov, Fieremans, 2020, Nilsson, Alexander, 2012, Nilsson, Lätt, Ståhlberg, van Westen, Hagslätt, 2012, Özarslan, Yolcu, Herberthson, Knutsson, Westin, 2018). Second, the extra-cellular component is modelled as a ball with isotropic diffusion. This assumption is not valid when there are coherently-oriented fibers (such as in the midline of the corpus callosum), where diffusion in the extra-axonal space can have a high anisotropy (Özarslan et al., 2011).

Limitations for the gray matter; it is assumed that the exchange between water environments is negligible. This assumption might be valid since previous studies have shown the exchange times in the white matter are of the order of seconds or longer (Bai, Li, Sun, Hsu, Liang, Basser, 2020, Lampinen, Szczepankiewicz, van Westen, Englund, C Sundgren, Lätt, Ståhlberg, Nilsson, 2017, Lasič, Nilsson, Lätt, Ståhlberg, Topgaard, 2011, Nilsson, Lätt, van Westen, Brockstedt, Lasič, Ståhlberg, Topgaard, 2013) which is much larger than the time-scales that the effects of restricted diffusion can be observed. However, exchange between compartments is likely to be non-negligible in the gray matter on the time-scale of the experiment (Badaut et al., 2011).

Limitations for both white matter and gray matter; first, at low frequencies, the time-dependency of diffusivity in the extracellular space can dominate over the time-dependency in the intra-axonal space (Burcaw, Fieremans, Novikov, 2015, Nilsson, Lasič, Drobnjak, Topgaard, Westin, 2017). Second, in our tissue model, we have neglected the distribution of restricted dimensions (e.g. range of soma sizes, axon diameters). However, adding extra parameters to the model to account for this will make the fitting unstable. Third, the effect of $T_{2}$ relaxation is not considered in our model which may result in bias in the estimation of the model parameters. In addition, the lack of cerebrospinal fluid (CSF) component in the model is another limitation of this work. An important limitation of the models used here is that they attempt to describe complex tissue with just a few parameters. For example, the stick model could be biased for neurite interpretation in the gray matter as well as white matter because of curvature (Özarslan et al., 2018). We should emphasize that our assumptions are based on healthy brain and in the presence of any pathology or other abnormalities these assumptions may be violated. For instance, if there is a lesion (i.e. a region of abnormal tissue), we would expect the lesion to appear also in the parametric maps derived from SANDI, but the neurobiological interpretation of the model parameters may be unclear and further histological evaluations/validation are needed to resolve any possible ambiguity. In the presence of a *distribution* of radii, we obtain and MR effective radius. For cylindrical restrictions, this is the sixth moment over the second moment of the distribution and it is therefore heavily weighted by the right tail of the distribution Veraart et al. (2020). For spherical restrictions, the MR effective radius is the seventh moment over the third moment, and will again be heavily tail-weighted. *Future directions*. The fitting method used in this work is a nonlinear least-square fit that can be replaced with new deep learning approaches to improve the quality of fit (Gibbons et al., 2019). From the acquisition perspective, the protocol that is used in this study is not optimized for the estimation of small sizes. Using a range of frequency spectra will help (Drobnjak et al., 2013). The protocol, used in this work, imposes a long acquisition time which can be minimized by optimizing the directions as well as the number of shells. In this paper, simple arithmetic averaging is used for powder averaging which can be replaced with some other techniques such as weighted averaging (Afzali, Knutsson, Özarslan, Jones, 2020, Knutsson, Andersson, Wiklund, 1999, Szczepankiewicz, Westin, Knutsson, 2017) to obtain a better orientation-invariant signal that improves the parameter estimates. With the development of imaging techniques we might be able to use these findings for clinical studies. The protocol used in this study is too long for clinical applications, in our future work we aim to optimize the acquisition toward clinical studies (Alexander, 2008, Lampinen, Szczepankiewicz, Mårtensson, van Westen, Hansson, Westin, Nilsson, 2020) to investigate if we can achieve the same results on clinical scanners with less strong gradients, and the concomitant increase in echo times and reduction in signal to noise ratio.

## 6 Conclusion

In this work, we have demonstrated key challenges and limitations in estimating sphere radius non-invasively in the human brain from diffusion MRI. Our simulations show the effect of Rician bias on the estimation of sphere radius and identified the lower bound limit of the sphere signal fraction and size that can be detected from the diffusion-weighted signal from both an SNR and empirical perspective. We showed that for small sphere signal fraction, i.e. <10%, this is a problem. However, we know from detailed microscopy of brain cortex (Beul, Hilgetag, 2019, Collins, Airey, Young, Leitch, Kaas, 2010), that in GM the soma signal fraction is on average > 20%. Therefore, reliable estimation of spherical compartment properties in GM is possible, while in WM it presents several challenges. The flat landscape of the fitting was also investigated. Using the ultra-strong gradients of the Connectom scanner, the diffusion signal in the white matter can be made sensitive to the axon diameter, and therefore the three-compartment model of stick + ball + sphere changes to cylinder + ball + sphere which has two time-dependencies, one for the diffusivity in the sphere and the other one for the diffusivity in the cylinder. Disentangling these two time-dependencies using only one sequence parameter (i.e., changing the frequency content of the encoding waveform) in the acquisition is challenging. Studying all these challenges prevents misinterpretation of the biased estimated parameters.

## Conflict of interest

MN declares ownership interests in Random Walk Imaging, and patent applications in Sweden (12504536 and 12504528), USA (61/642 594 and 61/642 589), and PCT (SE2013/050492 and SE2013/050493). Remaining authors declare no conflict of interest.

## Appendix A Supplementary materials

Supplementary Data S1 Supplementary Raw Research Data. This is open data under the CC BY license http://creativecommons.org/licenses/by/4.0/ Supplementary Data S1
