# Supplementary material for: SPHERIOUSLY? The challenges of estimating sphere radius non-invasively in the human brain from diffusion MRI
Source: Neuroimage. 2021 Aug 15;237:118183. doi: 10.1016/j.neuroimage.2021.118183 (PMC8285594; doi:10.1016/j.neuroimage.2021.118183)
Supplement: Supplementary Data S1 — Supplementary Raw Research Data. This is open data under the CC BY license http://creativecommons.org/licenses/by/4.0/ [file mmc1.pdf]

A. Supplementary Material

The effect of sphere size and signal fraction on exponent α in stick + ball + sphere model is shown in Fig S3. Fig S5 and S6 illustrate the results of fitting ball + stick + sphere and ball + cylinder + sphere model to the synthetic signal at three different noise levels (SNR = 20, 30, and 100). Fig S8 shows the estimated parameters of the stick + ball + sphere model on axial, coronal, and sagittal views of the *in vivo* brain image without applying the Gaussian kernel. In Fig S9, in addition to the parameters of the model we also estimate the standard deviation of the noise as it is explained in Section 3.1.

The reason for assuming an equal signal fraction for the ball compartment and the stick compartment was to simplify the simulation. We have also simulated the signal using the following parameters:

$f_{\text{sphere}} = 0.05,\ 0.1,\ 0.2,\ f_{\text{stick}} = 0.7,\ f_{\text{ball}} = 1 - f_{\text{stick}} - f_{\text{sphere}},\ D_{\text{in}}^{||} = 2\ \mu m^2/ms,\ D_{\text{ball}} = 0.6\ \mu m^2/ms,\ R_{\text{sphere}} = 1:1:10\ \mu m.$

where the stick signal fraction is much higher than the ball signal fraction but the results are not considerably different from those reported in Figure **??**, and therefore our conclusions remain unchanged.

Under the assumption that the diffusivity of the ball compartment is sufficiently high that its signal contribution is zero we ran the simulation for $f_{\text{ball}} = 0$ and $b \geq 3ms/\mu m^2$. Under this condition, the fitting is more stable. However, if the diffusivity of ball is small, we cannot exclude its contribution to the signal in the high b-value data. The low b-value measurements are obviously beneficial for the fitting of the ball compartment if its diffusivity is high and if the ball compartment has small diffusivity then it cannot be neglected in high b-value regime.

$f_{\text{sphere}} = 0.05,\ 0.1,\ 0.2,\ f_{\text{stick}} = 0.7,\ f_{\text{ball}} = 1 - f_{\text{stick}} - f_{\text{sphere}},\ D_{\text{in}}^{||} = 2\ \mu m^2/ms,\ R_{\text{sphere}} = 1:1:10\ \mu m.$

As far as healthy tissue is concerned, it has been estimated in vivo in the mouse brain (Nicholson and Hrabětová, 2017) that the tortuosity of the extracellular space in cortical GM is ~ 1.6, with $\sim 20\%$ volume fraction occupied by the extracellular space. In WM, the tortuosity may be higher, e.g. ~ 2.1, and the extracellular space may be lower, e.g. $\sim 5\%$. Assuming these values, the effective diffusivity is expected to be $D_0/tortuosity^2 \sim 0.7 - 1.2\ \mu m^2/ms$ going from $\sim 5\% - 20\%$ extracellular volume fraction, and assuming $D_0 = 3\ \mu m^2/ms$. These lead to a signal fraction of the extracellular ball compartment $< 0.5\%$ for $b \geq 3ms/\mu m^2$ (ignoring additional decay due to T_2 relaxation). We would safely expect this contribution to be negligible compared to the contribution of the stick/cylinder and sphere to the total signal at high b values, for the majority of conditions. Perhaps, in some specific pathological scenarios the ball contribution may be more relevant, and to include more challenging scenarios, we have considered the ball contribution in our fitting.

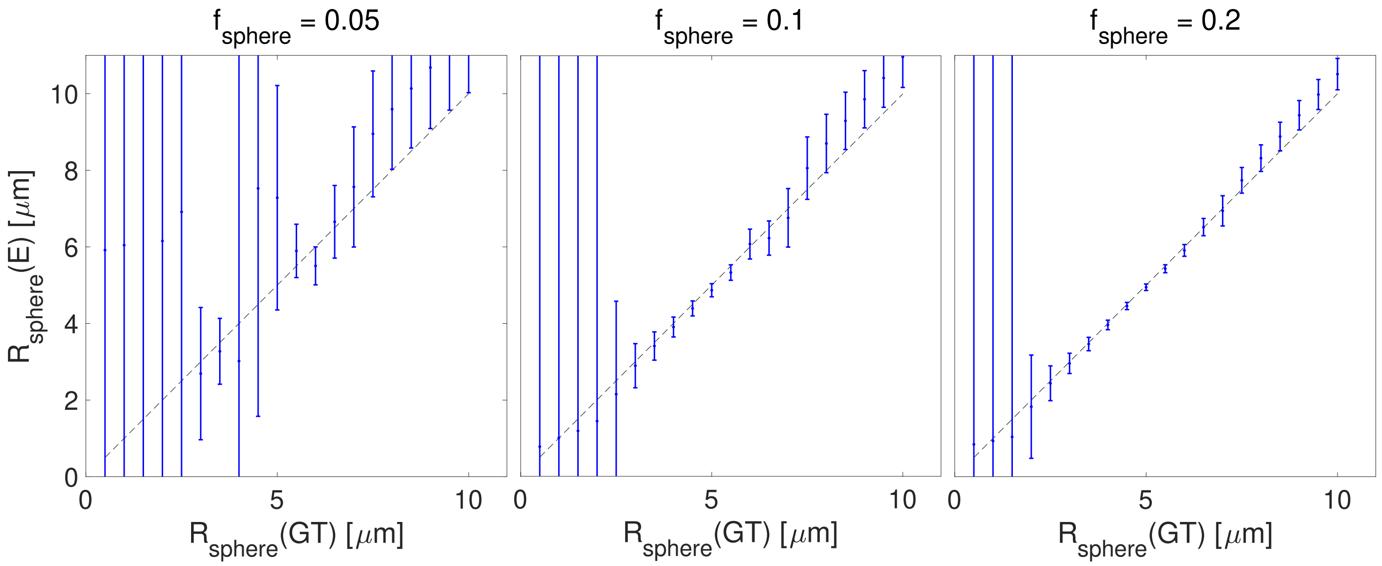

Figure S1: The results of fitting the sphere radius (stick + ball + sphere) for different sphere signal fractions (GT = Ground Truth and E = Estimated) in the presence of Rician noise with SNR = 50 (The diagonal black line is the line of identity and the error bars show the confidence interval).

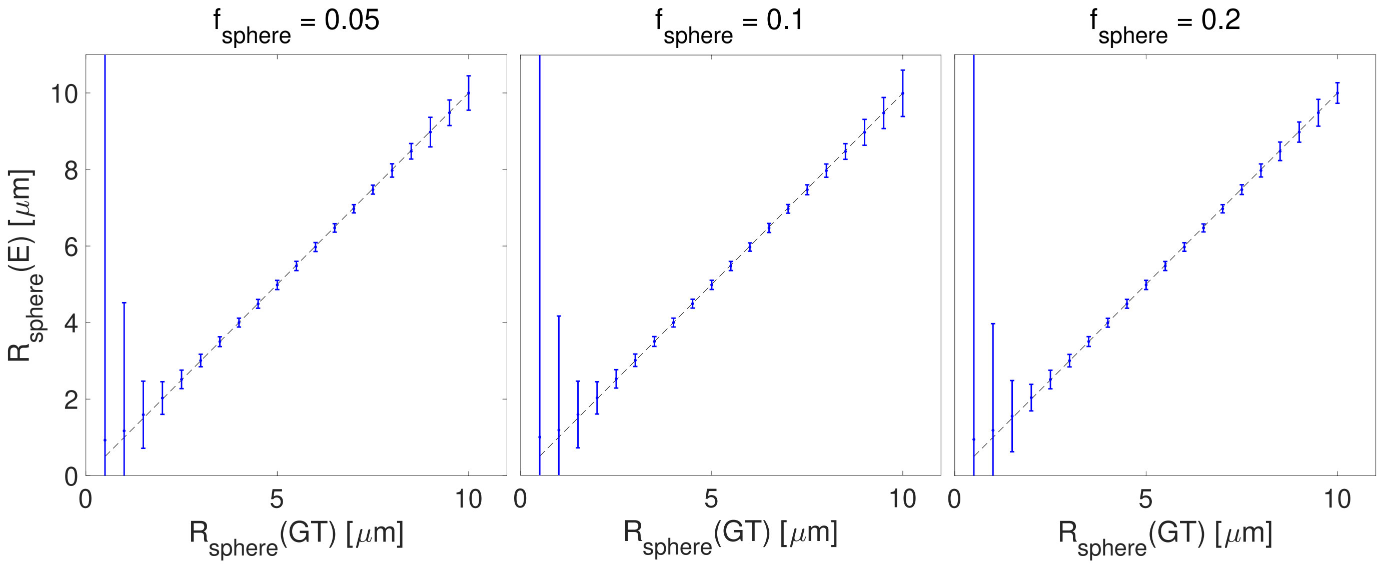

Figure S2: The results of fitting the stick + sphere model to the high b-value data ($b \geq 3ms/\mu m^2$) for different sphere signal fractions (GT = Ground Truth and E = Estimated) in the presence of Rician noise with SNR = 50 (The diagonal black line is the line of identity and the error bars show the confidence interval).

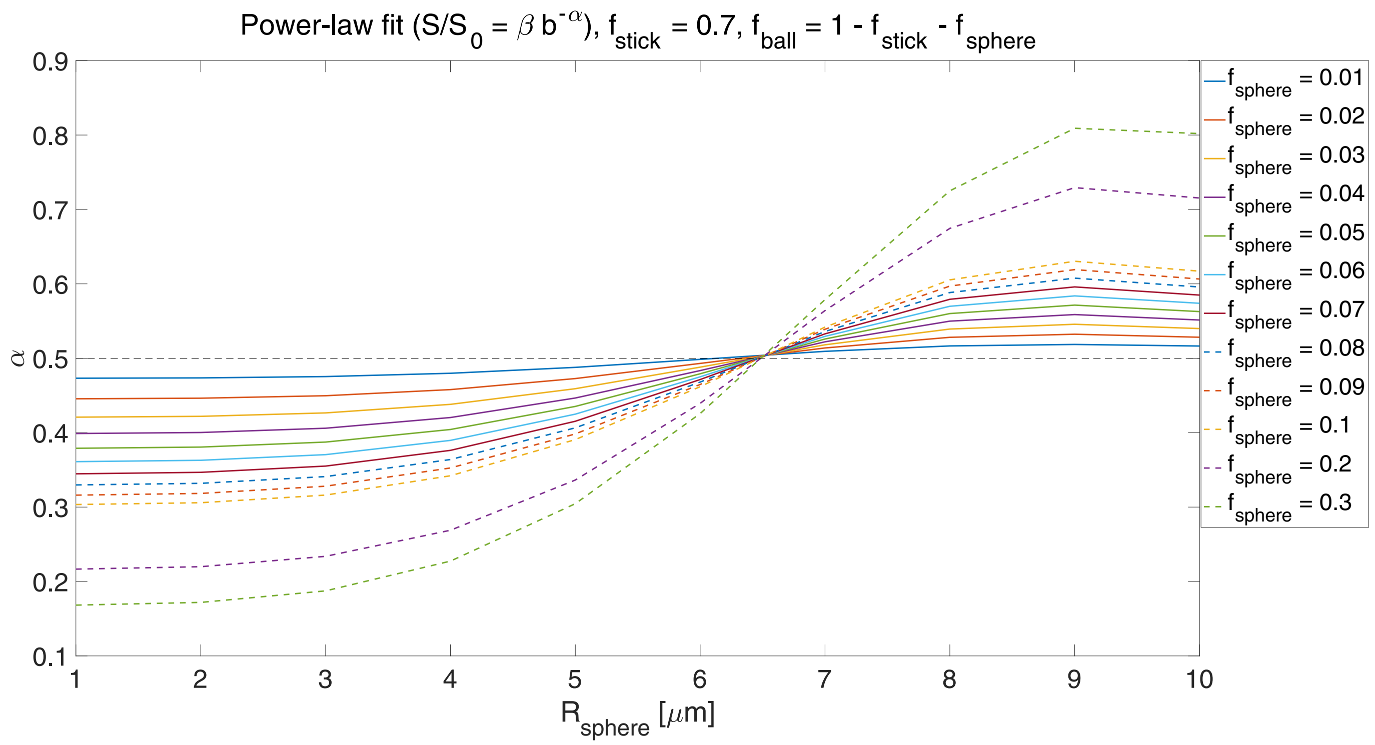

Figure S3: The effect of sphere size and signal fraction on exponent α. ($f_{\text{sphere}} = 0.01 : 0.01 : 0.1, 0.2 : 0.1 : 0.3$, $f_{\text{stick}} = 0.7$, $f_{\text{ball}} = 1 - f_{\text{stick}} - f_{\text{sphere}}$, $D_{\text{in}}^{||} = 2\ \mu m^2/ms$, $D_{\text{ball}} = 1\ \mu m^2/ms$, $R_{\text{sphere}} = 1 : 1 : 10\ \mu m$, $\delta = 29.65\ ms$, and $\Delta = 37.05\ ms$).

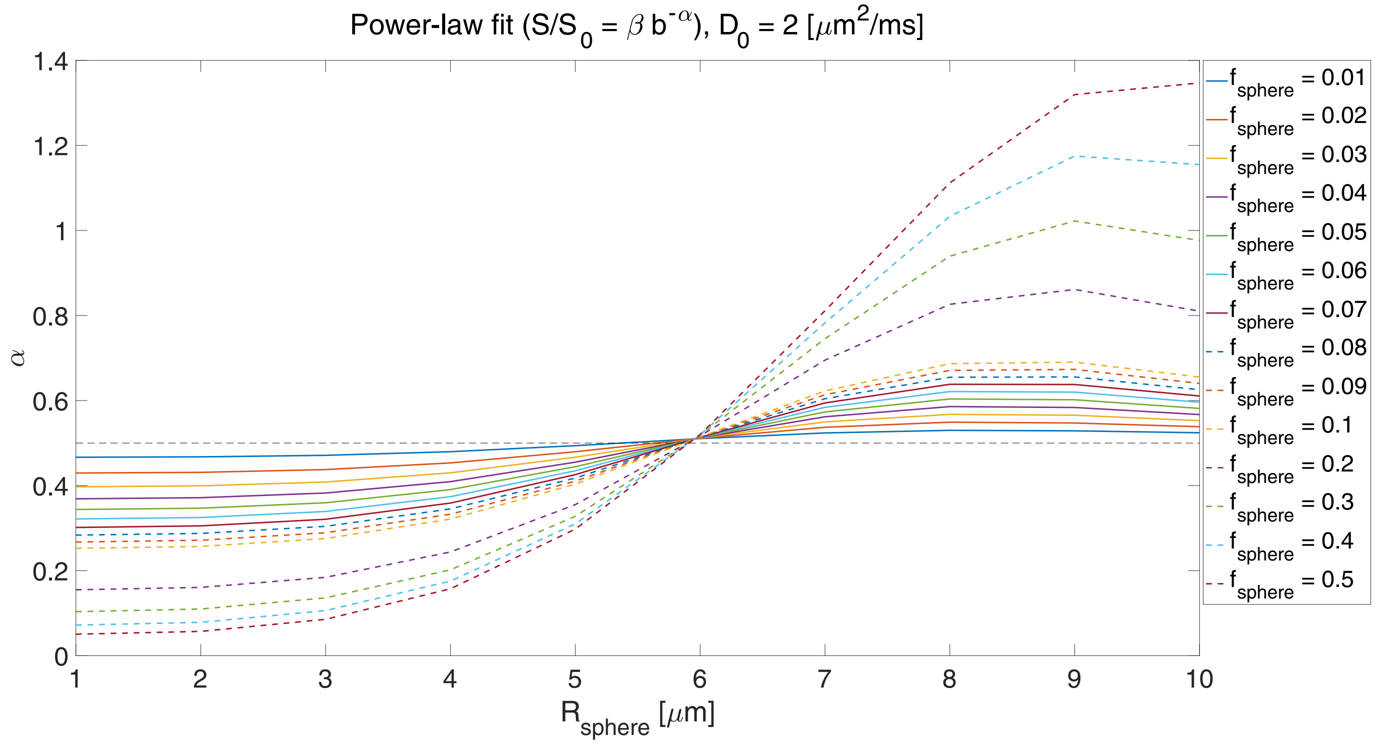

Figure S4: The effect of sphere size and signal fraction as well as D_0 on exponent α. ($f_{\text{sphere}} = 0.01 : 0.01 : 0.1, 0.2 : 0.1 : 0.5$, $f_{\text{ball}} = f_{\text{stick}} = (1 - f_{\text{sphere}})/2$, $D_{\text{in}}^{||} = 2\ \mu m^2/ms$, $D_{\text{ball}} = 2\ \ \mu m^2/ms$, $D_0 = 2\ \mu m^2/ms$, $R_{\text{sphere}} = 1 : 1 : 10\ \mu m$, $\delta = 29.65\ ms$, and $\Delta = 37.05\ ms$).

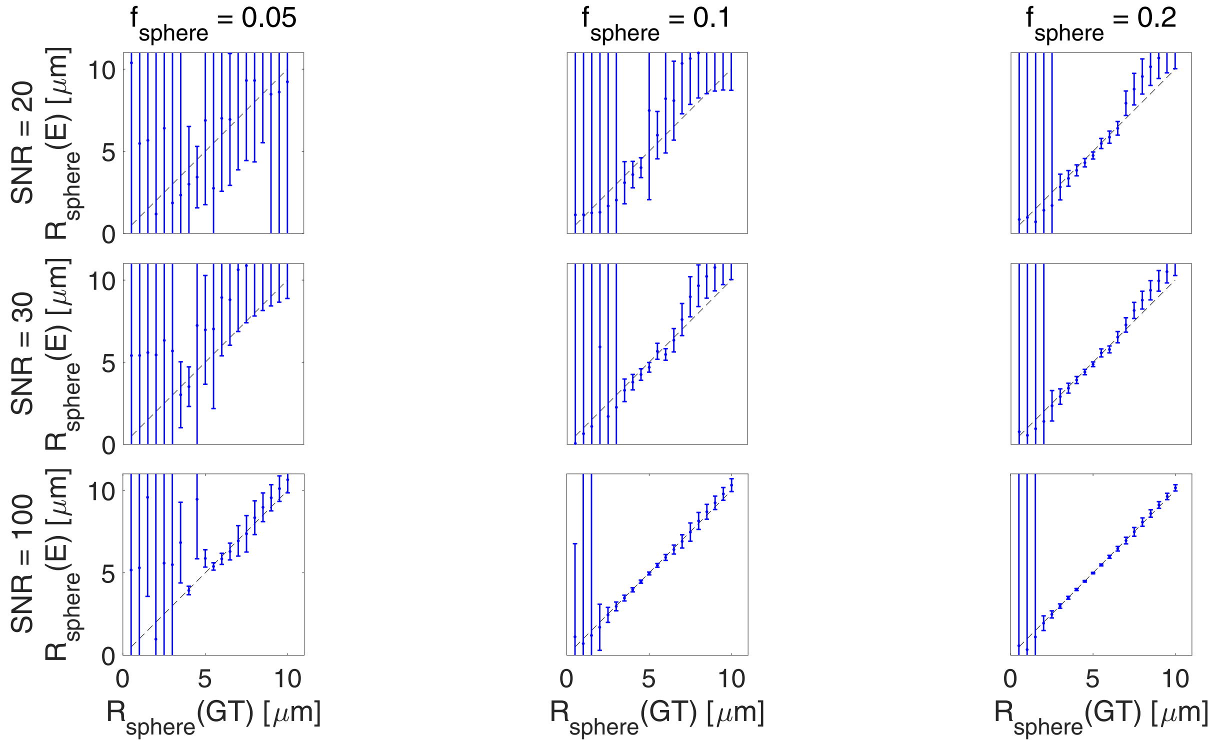

Figure S5: The results of fitting (ball + stick + sphere) the sphere radius for different sphere signal fractions and different noise floors (SNR = 20, 30, 100) (GT = Ground Truth and E = Estimated). The diagonal black line is the line of identity and the error bars show the confidence interval.

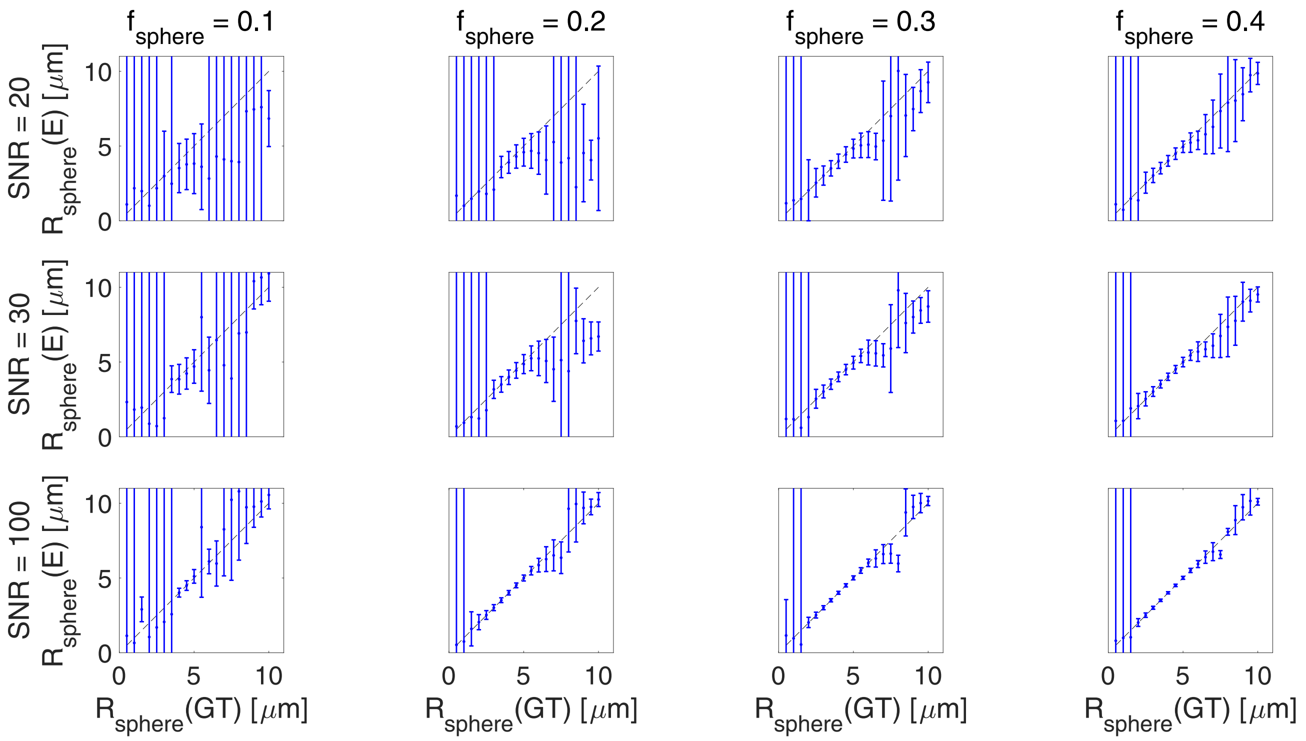

Figure S6: Estimated sphere radii versus the ground truth sphere radius values for cylinder + ball + sphere model (SNR = 20, 30, 100). (GT = ground truth and E = estimated). The diagonal black line is the line of identity and the error bars show the confidence interval.

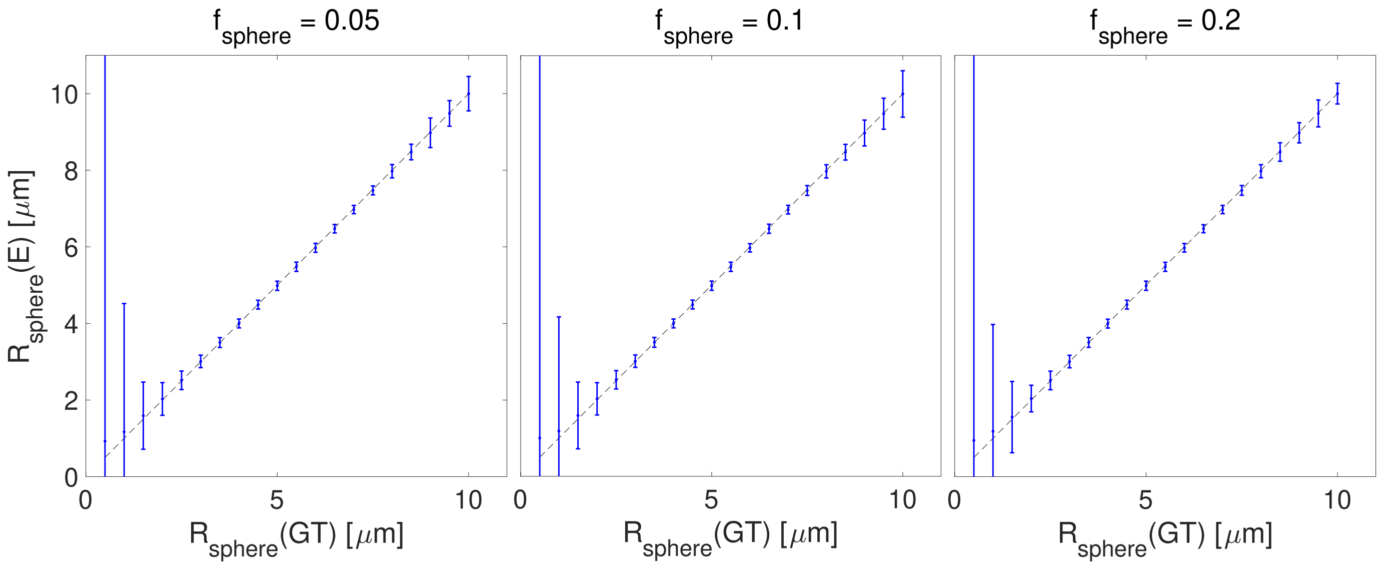

Figure S7: The results of fitting the stick + sphere model to the high b-value data ($b \geq 3ms/\mu m^2$) for different sphere signal fractions (GT = Ground Truth and E = Estimated) in the presence of Rician noise with SNR = 50 (The diagonal black line is the line of identity and the error bars show the confidence interval).

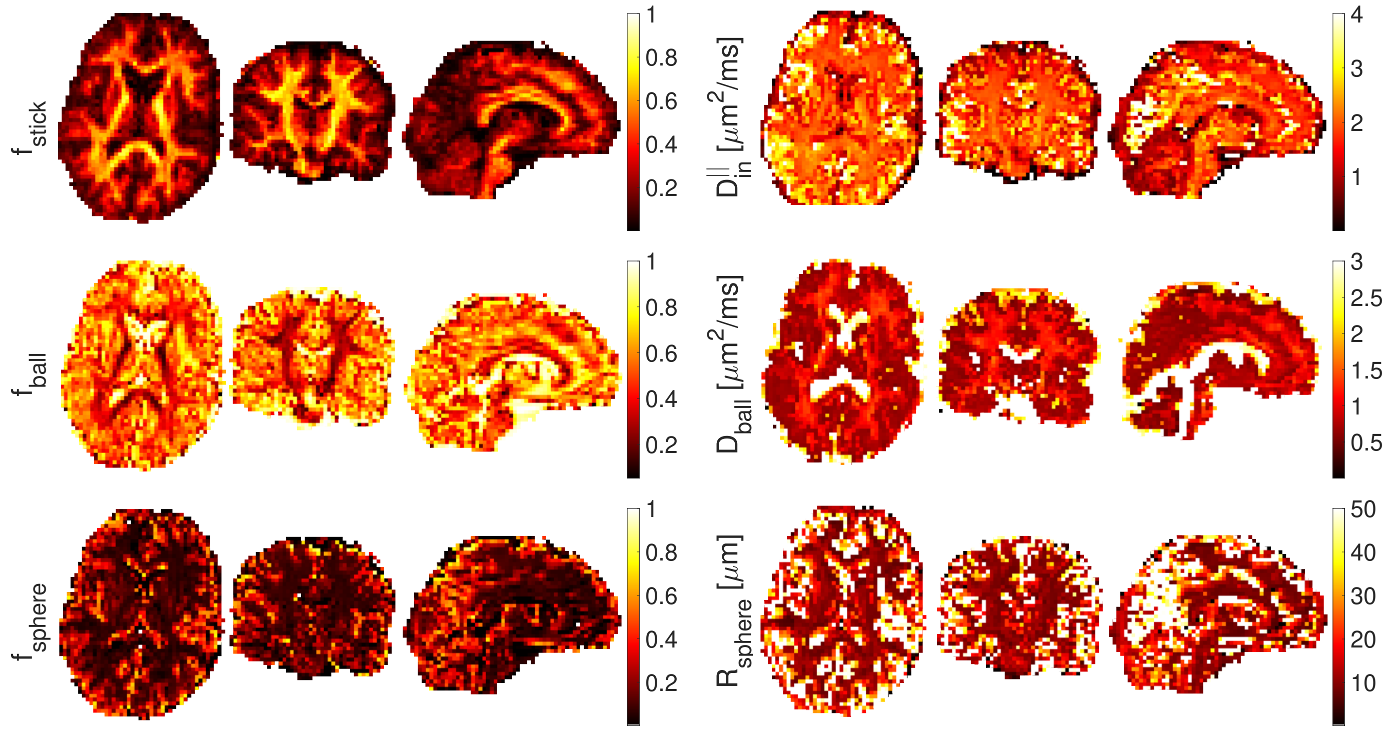

Figure S8: Estimated stick (f_{stick}), ball (f_{ball}), and sphere (f_{sphere}) signal fractions, intra-axonal parallel diffusivity ($D_{\text{in}}^{||}(\mu m^2/ms)$), extra-cellular diffusivity ($D_{\text{ball}}(\mu m^2/ms)$), and sphere radius ($R_{\text{sphere}}(\mu m)$) on axial, coronal, and sagittal views of the *in vivo* brain image.

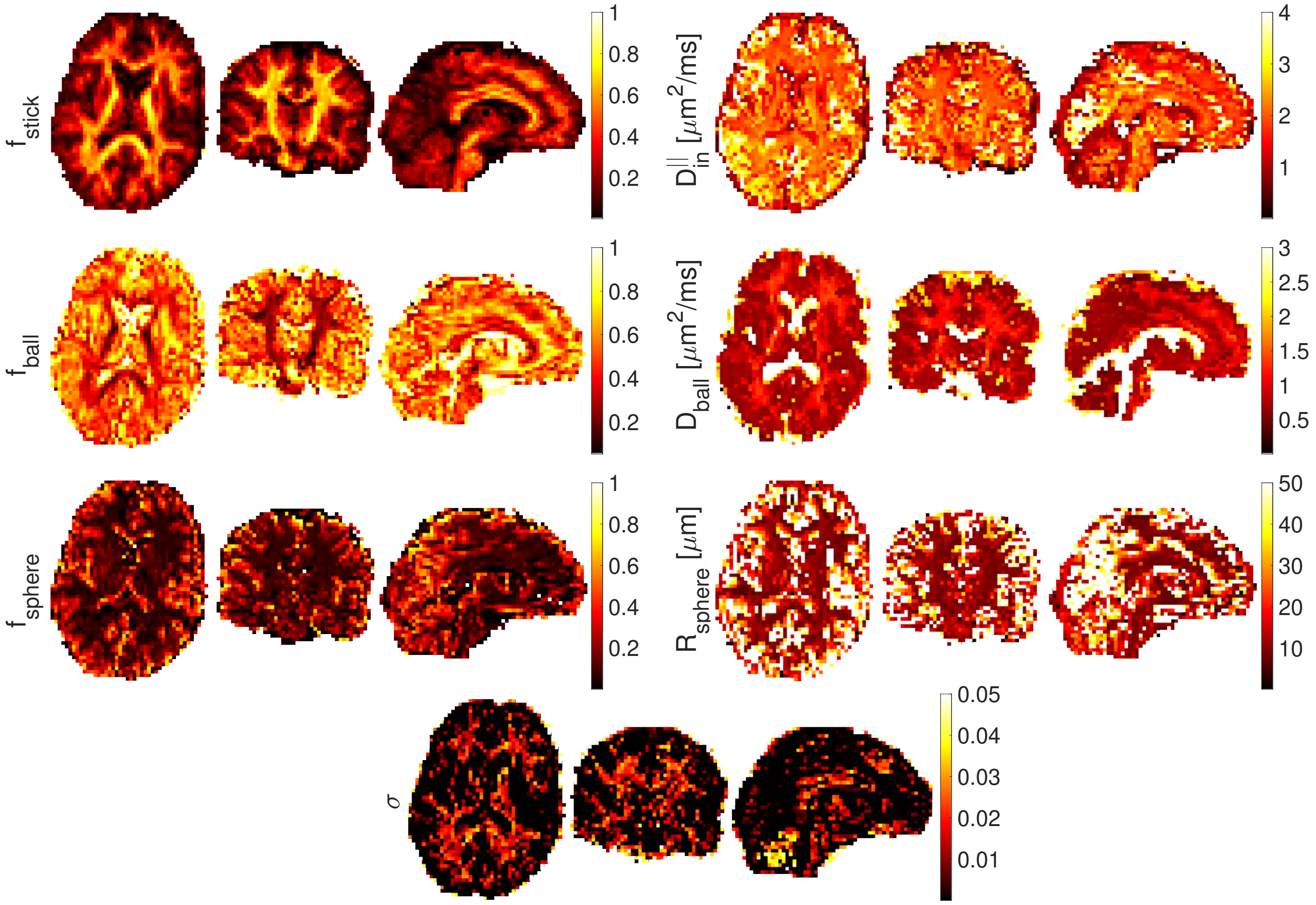

Figure S9: Estimated stick (f_{stick}), ball (f_{ball}), and sphere (f_{sphere}) signal fractions, intra-axonal parallel diffusivity ($D_{\text{in}}^{||}(\mu m^2/ms)$), extra-cellular diffusivity ($D_{\text{ball}}(\mu m^2/ms)$), sphere radius ($R_{\text{sphere}}(\mu m)$), and standard deviation of the noise (σ) on axial, coronal, and sagittal views of the *in vivo* brain image .
